# 4E-BPs require non-canonical 4E-binding motifs and a lateral surface of eIF4E to repress translation

**DOI:** 10.1038/ncomms5790

**Published:** 2014-09-02

**Authors:** Cátia Igreja, Daniel Peter, Catrin Weiler, Elisa Izaurralde

**Affiliations:** 1 Department of Biochemistry, Max Planck Institute for Developmental Biology, Spemannstrasse 35, 72076 Tübingen, Germany

## Abstract

eIF4E-binding proteins (4E-BPs) are a widespread class of translational regulators that share a canonical (C) eIF4E-binding motif (4E-BM) with eIF4G. Consequently, 4E-BPs compete with eIF4G for binding to the dorsal surface on eIF4E to inhibit translation initiation. Some 4E-BPs contain non-canonical 4E-BMs (NC 4E-BMs), but the contribution of these motifs to the repressive mechanism—and whether these motifs are present in all 4E-BPs—remains unknown. Here, we show that the three annotated *Drosophila melanogaster* 4E-BPs contain NC 4E-BMs. These motifs bind to a lateral surface on eIF4E that is not used by eIF4G. This distinct molecular recognition mode is exploited by 4E-BPs to dock onto eIF4E–eIF4G complexes and effectively displace eIF4G from the dorsal surface of eIF4E. Our data reveal a hitherto unrecognized role for the NC4E-BMs and the lateral surface of eIF4E in 4E-BP-mediated translational repression, and suggest that bipartite 4E-BP mimics might represent efficient therapeutic tools to dampen translation during oncogenic transformation.

## Abstract (web summary)

eIF4E-binding proteins (4E-BPs) are a conserved class of translational repressors that play essential roles in the regulation of protein expression. Here, Igreja *et al.* indentify non-canonical interactions between 4E-BPs and eIF4E that are required to effectively displace eIF4G and inhibit translation.

## Graphical abstract

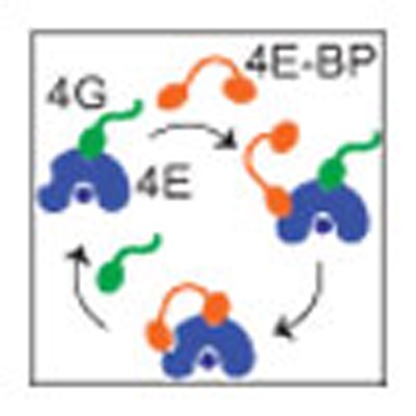

The regulation of protein synthesis at the initiation step is a widespread and reversible mechanism to control gene expression in eukaryotes^1^,^2^. During translation initiation, the small ribosomal subunit is recruited to mRNA by the eukaryotic initiation factor 4F (eIF4F) complex, which comprises the cap-binding protein eIF4E, the scaffolding protein eIF4G and the DEAD-box RNA helicase eIF4A. The eIF4E protein recognizes the mRNA m^7^GpppN cap structure and interacts with eIF4G, which promotes translation initiation via the recruitment of the 43S pre-initiation complex^1^. eIF4G binds eIF4E through a conserved motif (or canonical eIF4E-binding motif, C 4E-BM) of sequence TyrX_4_LeuΦ, where Φ is hydrophobic, and X is any amino acid^3^,^4^,^5^.

The assembly of the eIF4F complex is regulated by a diverse group of eIF4E-binding proteins (4E-BPs), which share a similar C TyrX_4_LeuΦ motif with eIF4G. Therefore, 4E-BPs bind to the same surface on eIF4E, sterically blocking its interaction with eIF4G and preventing translation initiation^4^,^5^,^6^,^7^. The association of 4E-BPs with eIF4E is reversible and regulated by phosphorylation. Unphosphorylated or hypophosphorylated 4E-BPs exhibit a high affinity for eIF4E and repress translation, whereas hyperphosphorylated 4E-BPs lose their affinity for eIF4E^2^,^8^,^9^.

At a functional level, 4E-BPs play essential roles in the control of translation during development and regulate neuronal plasticity by repressing translation at a global or message-specific level^9^,^10^,^11^,^12^,^13^,^14^. Through their inhibitory effect on translation, 4E-BPs negatively regulate cell proliferation and act as tumor suppressors^9^,^11^. However, the 4E-BP anti-oncogenic function is compromised in many tumors, resulting in increased eIF4E activity and protein synthesis, which is required for tumorigenic transformation^9^. Consequently, a detailed molecular understanding of the interaction between eIF4E and 4E-BPs is crucial to design or improve drugs that may be useful in pathological conditions in which eIF4E activity and global translation are upregulated^9^,^15^,^16^.

The C motifs of eIF4G and 4E-BPs adopt similar α-helical structures on binding to a conserved patch of hydrophobic residues on the dorsal side of the eIF4E cap-binding pocket^5^,^7^,^17^. Additional surfaces on eIF4E also contribute to the interaction with eIF4G as well as with a subset of 4E-BPs by binding to residues that are carboxy terminal to the C motifs, which contain NC 4E-BMs^17^,^18^,^19^,^20^. To date, NC motifs have only been identified and characterized in eIF4G, vertebrate 4E-BP1–3 and *D. melanogaster* CUP^17^,^19^,^21^,^22^,^23^. The NC motifs of 4E-BPs are not conserved between orthologous proteins across the animal kingdom. Therefore, it is not known whether all 4E-BPs contain NC motifs. Functionally, NC motifs have been proposed to play an auxiliary role by cooperating with their cognate C motifs to increase the binding affinity for eIF4E^17^,^19^,^20^,^22^.

The protein CUP is an insect-specific 4E-BP that controls the translation of maternal messenger RNAs during oogenesis and embryogenesis^21^,^24^,^25^,^26^. The crystal structure of *Dm* eIF4E bound to a CUP peptide containing the C and NC 4E-BMs revealed that both motifs adopt an α-helical conformation and contact two orthogonal surfaces on eIF4E^27^. The C 4E-BM binds to the conserved dorsal surface of eIF4E, as observed for the C motifs of eIF4G and 4E-BP1,2. The NC motif docks in an antiparallel fashion onto a lateral and conserved surface of eIF4E^27^.

A comparison of the *Dm* eIF4E–CUP complex with the structure of yeast eIF4E in complex with a fragment of eIF4G indicates that the NC motif of CUP and yeast eIF4G bind to partially overlapping surfaces on the lateral side of eIF4E^17^,^27^. Consequently, NC motifs could also contribute to the steric incompatibility with eIF4G and participate in the competition process. However, the contribution of NC motifs to the ability of 4E-BPs to displace eIF4G has not yet been elucidated.

To shed light on the role of NC motifs in 4E-BP-mediated translational repression, we investigated whether different *Dm* 4E-BPs contain NC motifs and how these motifs contribute to the displacement of eIF4G from eIF4E. We show that similar to CUP, Thor (ortholog of 4E-BP1–3) and 4E-T (4E-transporter) bind to eIF4E through a bipartite sequence that contains a C motif and a NC motif. The newly identified NC motifs in Thor and 4E-T share no sequence similarity with their vertebrate counterparts or with CUP. Nevertheless, these motifs share an overlapping lateral binding surface on eIF4E with the NC motif of CUP, which is required for the binding of 4E-BPs but not of eIF4G. The binding to an eIF4E surface that is not used by eIF4G allows 4E-BPs to dock onto preexisting eIF4E–eIF4G complexes to begin to displace eIF4G from the dorsal surface. Our data reveal a hitherto unrecognized diversity of NC motifs and establish the relevance of these motifs in the mechanism by which 4E-BPs repress translation. More generally, our data indicate that bipartite 4E-BP mimics have a competitive advantage over eIF4G and might represent potent repressors for the treatment of malignancies, in which eIF4E activity is upregulated.

## Results

### 4E-BPs bind to a lateral surface of eIF4E

**Figure 1:**
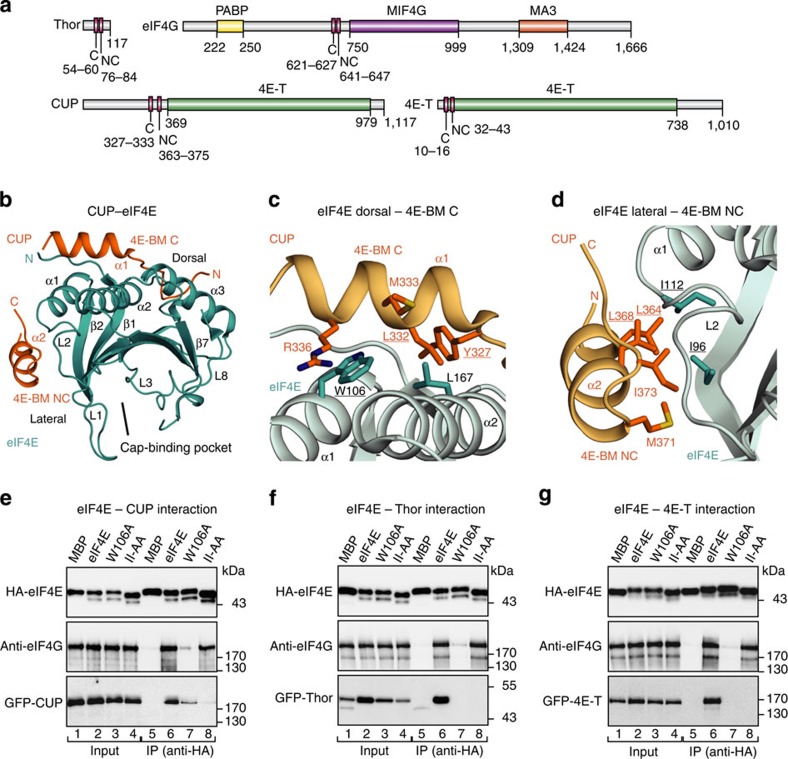
4E-BPs bind to a lateral surface of eIF4E. (**a**) Schematic representation of eIF4G and the 4E-BPs that were analyzed in this study. All the proteins contain a canonical (C) and a non-canonical (NC) 4E-BM. CUP and 4E-T contain a region with similarity to human 4E-transporter (4E-T) region. eIF4G contains a PABP-interacting region and MIF4G and MA3 domains. The amino-acid positions at the domain/motif boundaries are indicated below the protein outlines. (**b**) Cartoon representation of the overall structure of the eIF4E–CUP complex. eIF4E is shown in cyan and CUP in orange (PDB code 4AXG)^27^. Selected secondary structure elements are labeled in black for eIF4E and in orange for CUP. (**c**,**d**) Close-up views of the dorsal (**c**) and lateral (**d**) interfaces between eIF4E and CUP. Selected interface residues are shown as cyan and orange sticks for eIF4E and CUP, respectively. The eIF4E and CUP residues are labeled in black and orange, respectively, and underlined if they are mutated. (**e**–**g**) WB showing the interaction of HA–eIF4E (either WT or mutated) with GFP-tagged full-length 4E-BPs (CUP, Thor and 4E-T) and endogenous eIF4G. The size markers (kDa) are shown to the right of each panel. The original WB shown in this figure can be found in Supplementary Fig. 8.

To gain insight into the binding mode of different 4E-BPs to eIF4E, we compared the interaction of *Dm* CUP, Thor and 4E-T with *Dm* eIF4E (Fig. 1a). In coimmunoprecipitation and pull-down assays, we confirmed that all the proteins interacted with endogenous eIF4E in *Dm* Schneider (S2) cells (Supplementary Fig. 1a–e).

*Dm* CUP interacts with eIF4E through C and NC motifs^27^. In particular, the CUP residues Tyr327, Leu332, Met333 and Arg336 in the C motif interact with residues on the dorsal surface of eIF4E, including Trp106 and Leu167 (Fig. 1b,c and Supplementary Fig. 2a,b). In addition, the CUP residues Leu364, Leu368, Met371 and Ile373 in the NC motif contact a eIF4E lateral surface that is centered at residues Ile96 and Ile112 (Fig. 1b,d and Supplementary Fig. 2a,b)^27^.

To determine whether Thor, 4E-T and eIF4G also recognize the lateral surface of eIF4E, we substituted residues Ile96 and Ile112 with Ala (eIF4E mutant II-AA) and performed coimmunoprecipitation assays in S2 cells. As a control, we used an eIF4E mutant with a Trp106Ala substitution (W106A) on the dorsal binding surface, because this substitution abolishes the binding of CUP and eIF4G to eIF4E^21^,^28^,^29^. As expected, the W106A substitution strongly reduced the binding of eIF4E to endogenous eIF4G and to all three of the 4E-BPs (Fig. 1e–g, lanes 7). By contrast, the II-AA mutations disrupted the association of eIF4E with CUP, Thor and 4E-T but not with eIF4G (Fig. 1e–g, lanes 8). Thus, in contrast to eIF4G, 4E-BPs recognize and depend on the lateral surface to efficiently bind to eIF4E in cell lysates, in which eIF4G (or other 4E-BPs) is also present.

### Identification of NC 4E-BMs in Thor and 4E-T

The immunoprecipitation assays shown in Fig. 1e–g indicate that similar to CUP, Thor and 4E-T contain NC motifs that interact with the lateral binding surface of eIF4E. In human 4E-BP1,2, the NC IPGVTS/T motif (located C-terminally to the C motif), increases the binding affinity of the proteins for eIF4E by approximately three orders of magnitude^19^,^22^. However, the IPGVTS/T motif is not conserved across the animal kingdom (Supplementary Fig. 2c). Nevertheless, several hydrophobic residues are present in the corresponding region in *Dm* Thor (residues Pro76–Pro84; Supplementary Fig. 2c).

**Figure 2:**
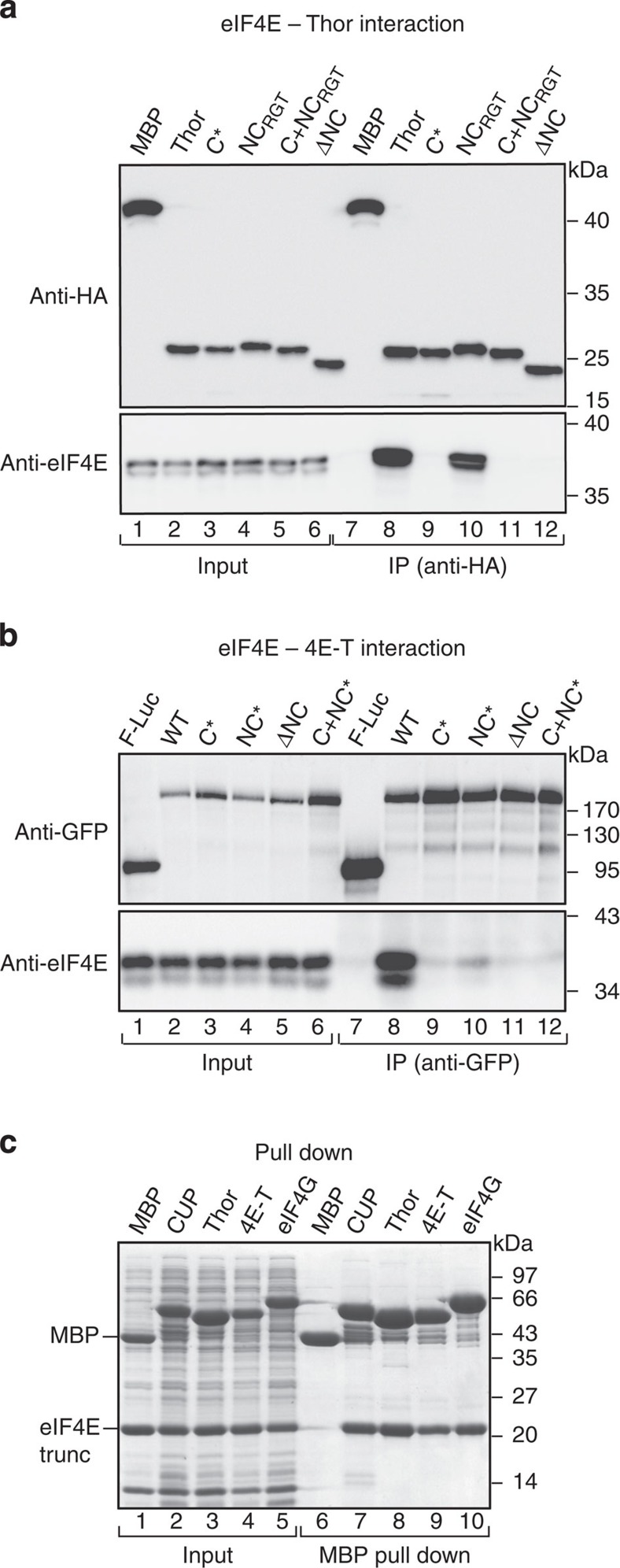
Identification of non-canonical motifs in Thor and 4E-T. (**a**) WB showing the interaction of HA-Thor (full length, either WT or mutated) with endogenous eIF4E in S2 cells. The proteins were immunoprecipitated using anti-HA antibodies. The inputs (1%) and immunoprecipitates (30%) were analyzed by WB using anti-HA and anti-eIF4E antibodies. The original WB shown in this panel can be found in Supplementary Fig. 8. (**b**) WB showing the interaction of GFP–4E-T (full length, WT or mutated) with endogenous eIF4E. The proteins were immunoprecipitated using anti-GFP antibodies. The inputs (1%) and immunoprecipitates (30%) were analyzed by WB using anti-GFP and anti-eIF4E antibodies. The original WBs can be found in Supplementary Fig. 8. (**c**) MBP pull down showing the interaction of His_6_-tagged eIF4E (residues 69–248, trunc) with MBP-tagged 4E-BP C+NC fragments (see Supplementary Table 1) and eIF4G (residues 578–650). All the fragments contained, in addition, a C-terminal GB1 tag. The input samples (10%) and bound fractions (15%) were analyzed using SDS–PAGE followed by Coomassie blue staining. The size markers (kDa) are shown to the right of each panel.

To determine whether the Thor residues 76–84 constitute a NC 4E-BMs, we substituted Cys78, Leu79 and Leu80 with alanine (NC*) or deleted the motif (ΔNC, Supplementary Table 1). In the coimmunoprecipitation assays, the deletion of the Thor residues 76–84 abolished the interaction with eIF4E (Fig. 2a, lane 12), whereas the alanine substitutions decreased the eIF4E binding (Supplementary Fig. 1d, lane 9, NC*). By contrast, the substitution of the flanking residues Arg81, Gly82 and Thr83 by alanine was ineffective (Fig. 2a, lane 10). As a control, amino-acid substitutions in the C motif (C*, Supplementary Table 1) also disrupted the interaction with eIF4E (Fig. 2a and Supplementary Fig. 1d). Thus, the interaction of Thor with eIF4E requires both a C and a downstream NC motif in cell lysates.

In human 4E-T, sequences downstream of the C motif also contribute to the interaction with eIF4E^30^. Again, these sequences are not conserved in insects (Supplementary Fig. 2d). Nevertheless, based on the observation that in CUP and Thor, the NC motifs are located ~15–29 residues from the C motifs, are hydrophobic and, in the case of CUP, exhibit helical propensity, we inspected the *Dm* 4E-T sequence for motifs that fulfill these criteria. We identified a region in the insect 4E-T (residues 32–43) that could contain a potential NC motif and is located at a similar position as is the motif in the human protein (Supplementary Fig. 2d). In the coimmunoprecipitation assays, alanine substitutions or deletions of various residues in this motif (Supplementary Table 1) caused a drastic reduction in the 4E-T binding to eIF4E (Fig. 2b, lanes 10 and 11, and Supplementary Fig. 1e), similar to the disruption of the C motif (C*, Fig. 2b, lane 9, and Supplementary Fig. 2d). Thus, a NC 4E-BMs is also present in the *Dm* 4E-T that is conserved in *Drosophila* species.

### 4E-BPs and eIF4G display similar affinities for eIF4E

Next, we compared the binding efficiencies of the minimal eIF4E-binding regions of the 4E-BPs (C+NC, Supplementary Table 1) in pull-down assays. These regions were expressed with an amino-terminal MBP-tag and a C-terminal GB1-tag^31^. In parallel, we analyzed the minimal eIF4E-binding fragment of eIF4G (residues 578–650), which includes the C motif and the SDVVL motif that was identified in *Hs* eIF4G (corresponding to *Dm* VKNVSI, Supplementary Fig. 2e), which plays an auxiliary function in stabilizing the eIF4G interaction with eIF4E^23^. The bipartite C+NC regions of the three 4E-BPs and the eIF4G fragment pulled down the purified eIF4E at comparable levels (Fig. 2c).

To obtain information on the affinities and thermodynamic parameters, we performed isothermal titration calorimetry (ITC) experiments. The bipartite regions of all three of the 4E-BPs and eIF4G exhibited comparable binding affinities for eIF4E, with dissociation constants (*K*_D_s) in the nanomolar range (Table 1 and Supplementary Fig. 3). The *K*_D_ values obtained for *Dm* Thor and eIF4G are comparable to those that have been reported for the human proteins^5^,^19^,^22^,^23^,^32^. Notably, although the binding of all proteins to eIF4E is enthalpically driven, the entropic penalties differ between these proteins, suggesting differences in the binding mechanisms. In particular, the interaction between CUP and eIF4E displayed the highest entropic penalty, which is indicative of a lower degree of conformational freedom in the bound state. Thus, CUP may undergo larger disorder-to-order transitions on binding, which is consistent with the formation of two α-helices^27^. 4E-T and Thor exhibited lower entropic penalties, suggesting a more dynamic conformation in the bound state.

To understand the contribution of the NC 4E-BMs to the affinity of 4E-BPs for eIF4E, we analyzed the binding of 4E-BP peptides containing only the C motifs or the complementary sequences comprising the linker (L) region between the two motifs and the NC motif (L+NC, Supplementary Fig. 4 and Supplementary Table 1). The affinities of the C motifs in isolation were one to three orders of magnitude lower than the C+NC peptides, indicating that the NC motifs contribute significantly to the overall affinity. Interestingly, the C motifs of all three 4E-BPs exhibited significant differences in binding affinities, with the affinity of the 4E-T peptide being approximately one and two orders of magnitude higher than those of the CUP and Thor peptides, respectively (Table 1 and Supplementary Fig. 4). The differences between 4E-BPs were more pronounced for the L+NC peptides, because only the CUP peptide interacted with eIF4E at detectable levels. The binding of the CUP peptide (L+NC) was enthalpically driven, with a *K*_D_ comparable to that of the C motif. These results indicate a similar contribution to the energetics of binding by the C and NC motifs of CUP.

Finally, we determined the affinities of the bipartite peptides (C+NC) for the eIF4E II-AA mutant. The affinities of CUP and Thor peptides were reduced by one and two orders of magnitude, respectively (Table 1 and Supplementary Fig. 5). In contrast, 4E-T binding was not significantly affected perhaps reflecting the higher affinity of its C 4E-BM. Similarly, the mutations in the lateral surface of eIF4E did not affect eIF4G binding.

We conclude that although 4E-BPs and eIF4G display similar affinities for eIF4E, they use different binding modes. These differences can be mainly attributed to the linker regions and the NC motifs, consistent with their sequence diversity, although differences in affinities for the C motifs were also detected. Moreover, the results of the ITC experiments also indicate that the affinity of 4E-BPs for eIF4E results from synergistic effects between the C and NC motifs.

### 4E-BP NC motifs are sufficient to bind eIF4E

**Figure 3:**
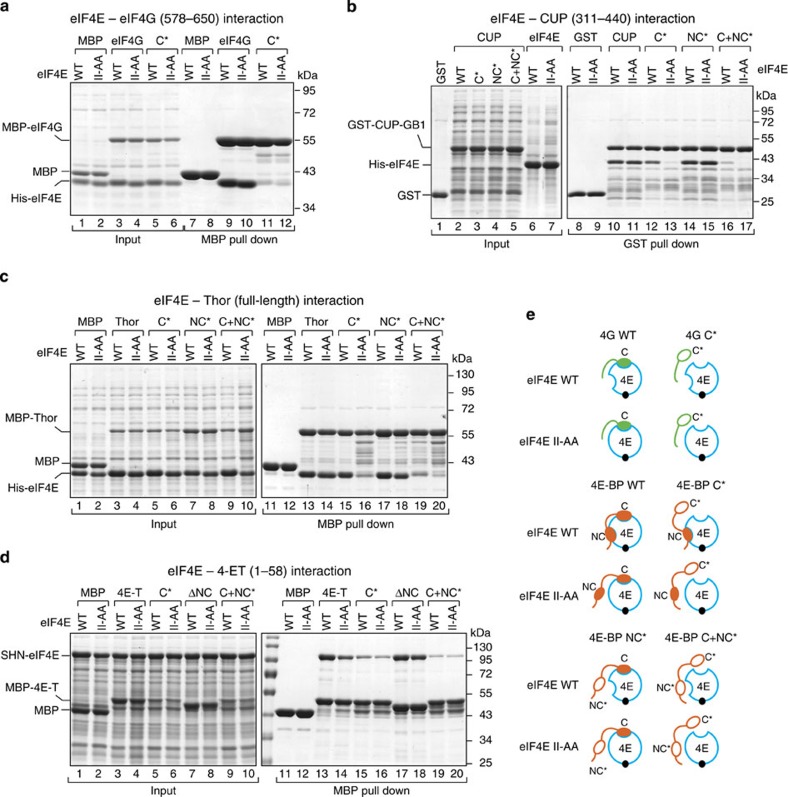
4E-BPs interact with eIF4E using a bipartite-binding mechanism. (**a**) MBP pull-down assay showing the interaction of His_6_-eIF4E (full length, either WT or II-AA mutant) and MBP-eIF4G (residues 578–650; either WT or canonical 4E-BM mutant (C*)). The input (25%) and bound fractions (50%) were analyzed by SDS–PAGE followed by Coomassie blue staining. (**b**) GST pull-down assay showing the interaction of His_6_-eIF4E (WT or II-AA mutant) and GST-CUP-GB1 (residues 311–440, WT or mutated in the canonical motif (C*), non-canonical motif (NC*) or both motifs (C+NC*)). (**c**) MBP pull down showing the association of MBP-Thor (full length, WT or 4E-BM mutants) with His_6_-eIF4E (WT or II-AA mutant). The samples were analyzed as described in **a**. (**d**) MBP pull-down assay showing the interaction of MBP–4E-T (fragment 1–58, WT or the indicated mutants) with eIF4E (WT or II-AA mutant). eIF4E was expressed with a tag consisting of the streptavidin-binding peptide (strep), His_6_ and the NusA protein (SHN tag). (**e**) Schematic representation of the different eIF4E–4E-BPs and eIF4E–eIF4G complexes that were analyzed in the pull-down assays. 4E, eIF4E (blue circle). Black circle, m^7^GTP-cap structure. The 4E-BPs are shown in orange and the eIF4G in green. The asterisks indicate mutations in the corresponding motifs. These mutations are described in Supplementary Table 1.

To further analyze the binding modes of the 4E-BPs and eIF4G to eIF4E, we performed pull-down assays with recombinant proteins that were expressed in *Escherichia coli*. In contrast to the experiments in cell lysates, the *in vitro* pull-down assays allowed us to investigate the interactions of the individual proteins in the absence of other 4E-BPs, which could compete for binding and could obscure the interpretation of the results. We tested recombinant fragments of eIF4G, CUP and 4E-T and full-length Thor for binding to either the eIF4E wild-type (WT) or II-AA mutant (that is, with a disrupted lateral surface). eIF4G and the 4E-BPs pulled down comparable amounts of WT and mutant eIF4E (Fig. 3a, lanes 9 and 10; Fig. 3b, lanes 10 and 11; and Fig. 3c,d, lanes 13 and 14), indicating that these proteins interact with the eIF4E mutant lacking a functional lateral binding surface *in vitro*. The results obtained *in vitro* contrast with the observation that the 4E-BPs did not interact with the II-AA mutant in cell lysates (Fig. 1e–g). One possible explanation for this difference is that cell lysates contain eIF4G, which blocks the dorsal surface of eIF4E, leaving only the lateral surface available for 4E-BPs. If the lateral surface is in addition mutated, then 4E-BPs may not be able to interact with eIF4E and displace bound eIF4G (see below).

The interaction of eIF4G and 4E-BPs with the eIF4E II-AA mutant is most likely mediated by their C motifs that bind to the dorsal surface of eIF4E. To confirm this assumption, we introduced mutations in the C motifs (C* mutants, Supplementary Table 1). Substitutions in the C motif of eIF4G abolished its interaction with either WT or mutant eIF4E (Fig. 3a, lanes 11 and 12). By contrast, the equivalent substitutions in the C motifs of 4E-BPs did not prevent their binding to eIF4E, reflecting a truly bipartite-binding mode (Fig. 3b, lane 12, and Fig. 3c,d, lane 15). However, the CUP and Thor C* mutants were strongly impaired in their binding to the eIF4E II-AA mutant (Fig. 3b, lane 13; Fig. 3c, lane 16), indicating that the C* mutants use the lateral surface of eIF4E. The 4E-T C* mutant showed reduced binding to both WT and mutant eIF4E (Fig. 3d, lanes 15 and 16).

Substitutions in the NC motifs (NC*) did not prevent the interaction of 4E-BPs with either WT or mutant eIF4E, most likely because the C motifs are sufficient for binding (Fig. 3b, lanes 14 and 15; Fig. 3c,d, lanes 17 and 18). The interaction of the three 4E-BPs with WT eIF4E was strongly reduced when the two motifs were mutated (C+NC*, Fig. 3b, lane 16, Fig. 3c,d, lanes 19). Remarkably, some residual binding to eIF4E was observed. These results suggest that the linker regions between the motifs in CUP and 4E-T and additional residues in Thor (which was full length) contact eIF4E and contribute to the interaction. The results obtained for the Thor NC* and C+NC* mutants were confirmed using a mutant with a deleted NC motif (ΔNC, Supplementary Fig. 6a).

Collectively, our results indicate that 4E-BPs interact with eIF4E using a bipartite-binding mode and recognize a lateral surface on eIF4E that is not used by eIF4G. Two main observations support these conclusions. First, mutations in the C motifs abolish the interaction of eIF4G but not of 4E-BPs with eIF4E. Second, mutations on the lateral surface of eIF4E abolish or reduce the binding of 4E-BPs to eIF4E when their binding to the dorsal surface is also compromised. Our results further indicate that the eIF4G residues downstream of the C motif, including the VKNVSI motif, do not use the binding surface centered at residues Ile96 and Ile112 and are not sufficient for binding to eIF4E when the C motif is mutated, which is in agreement with the proposed auxiliary role of these sequences^23^. Finally, it is important to note that although mutations in the C motifs of Thor and 4E-T do not disrupt binding to eIF4E, a deletion of the C motif prevents binding (L+NC peptides, see ITC experiments). These results suggest that mutations in the C motifs of these proteins do not completely abolish binding to the eIF4E dorsal surface, or that the formation of an α-helical structure (which is likely maintained in the mutants) is indirectly required to facilitate the binding of the linker region and NC motifs.

### 4E-BPs use the eIF4E lateral surface to compete with eIF4G

The observation that 4E-BPs can bind to the eIF4E II-AA mutant *in vitro* (that is, in the absence of competition) but not in cell lysates (that is, in the presence of eIF4G) suggests that 4E-BPs are not able to compete with eIF4G for binding to eIF4E when the lateral binding surface is disrupted.

**Figure 4:**
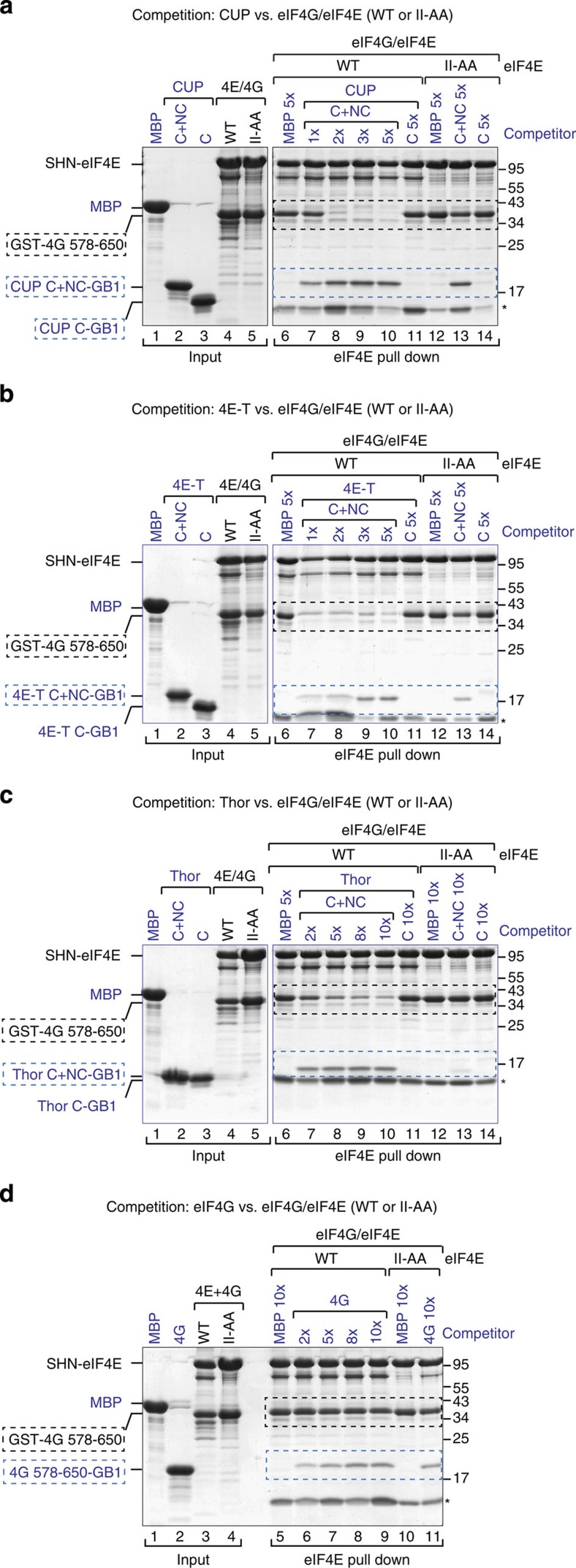
4E-BPs require binding to the lateral surface of eIF4E to compete with eIF4G. (**a**–**d**) Purified eIF4E–eIF4G complexes (2 μM) containing SHN-tagged eIF4E (full length, WT or II-AA mutant) and GST-eIF4G (residues 578–650) were incubated with increasing amounts of CUP (**a**), 4E-T (**b**), Thor (**c**) and eIF4G (**d**) peptides fused C terminally to GB1. The 4E-BP peptides contained the canonical and non-canonical motifs (C+NC) or only the C and are described in Supplementary Table 1. MBP served as a negative control. The proteins that were bound to eIF4E were pulled down using Strep-Tactin beads. The competitor peptides are labeled in blue, and their positions are highlighted by blue, dashed boxes. The black dashed boxes indicate the position of preassembled GST-eIF4G. Numbers above the lanes indicate fold molar excess of the competitor peptides. The corresponding quantification of the competition assays is shown in Supplementary Fig. 7a–d (*n*=2). Asterisks indicated a contaminant protein.

To further investigate the role of the lateral binding surface of eIF4E in the competition mechanism, we performed competition assays using preassembled eIF4E–eIF4G complexes containing either eIF4E WT or the II-AA mutant and GST-tagged eIF4G (residues 578–650). eIF4G formed stable complexes both with WT and mutant eIF4E (Fig. 4a–c, lanes 4 and 5, respectively). These preassembled eIF4E–eIF4G complexes were challenged with increasing amounts of peptides containing the C and NC (C+NC) motifs of 4E-BPs or the same eIF4G fragment. Proteins that were associated with eIF4E were recovered by eIF4E pull-down assays.

The CUP, 4E-T and Thor C+NC peptides displaced eIF4G from the complex and associated with eIF4E (Fig. 4a–c, lanes 7–10 versus 6, Supplementary Figs 6b–d and 7a–c). The CUP and 4E-T peptides were able to effectively displace eIF4G when present at two- and onefold molar excess, respectively. Under the same conditions, the 4E-BP C+NC peptides did not efficiently displace eIF4G from complexes that contained the eIF4E II-AA mutant (Fig. 4a–c, lanes 13 versus 12, Supplementary Figs 6b–d and 7a–c). Thus, binding to the lateral surface is required for 4E-BPs to effectively compete with eIF4G. In agreement with this conclusion, peptides containing only the 4E-BP C motifs did not displace eIF4G from eIF4E, although they were tested at the highest molar concentration (Fig. 4a–c, lanes 11 versus 10 and Supplementary Fig. 7a–c).

In striking contrast to the 4E-BP peptides, the eIF4G peptide hardly competed with GST-eIF4G for binding to eIF4E, irrespective of whether eIF4E was WT or mutated (Fig. 4d, lanes 5–11, Supplementary Figs 6b and 7d). Mechanistically, our results indicate that 4E-BPs are more efficient competitors than is eIF4G and must bind to the lateral surface of eIF4E to effectively displace eIF4G from preassembled eIF4E–eIF4G complexes.

### 4E-BPs use the NC motifs to compete with eIF4G

**Figure 5:**
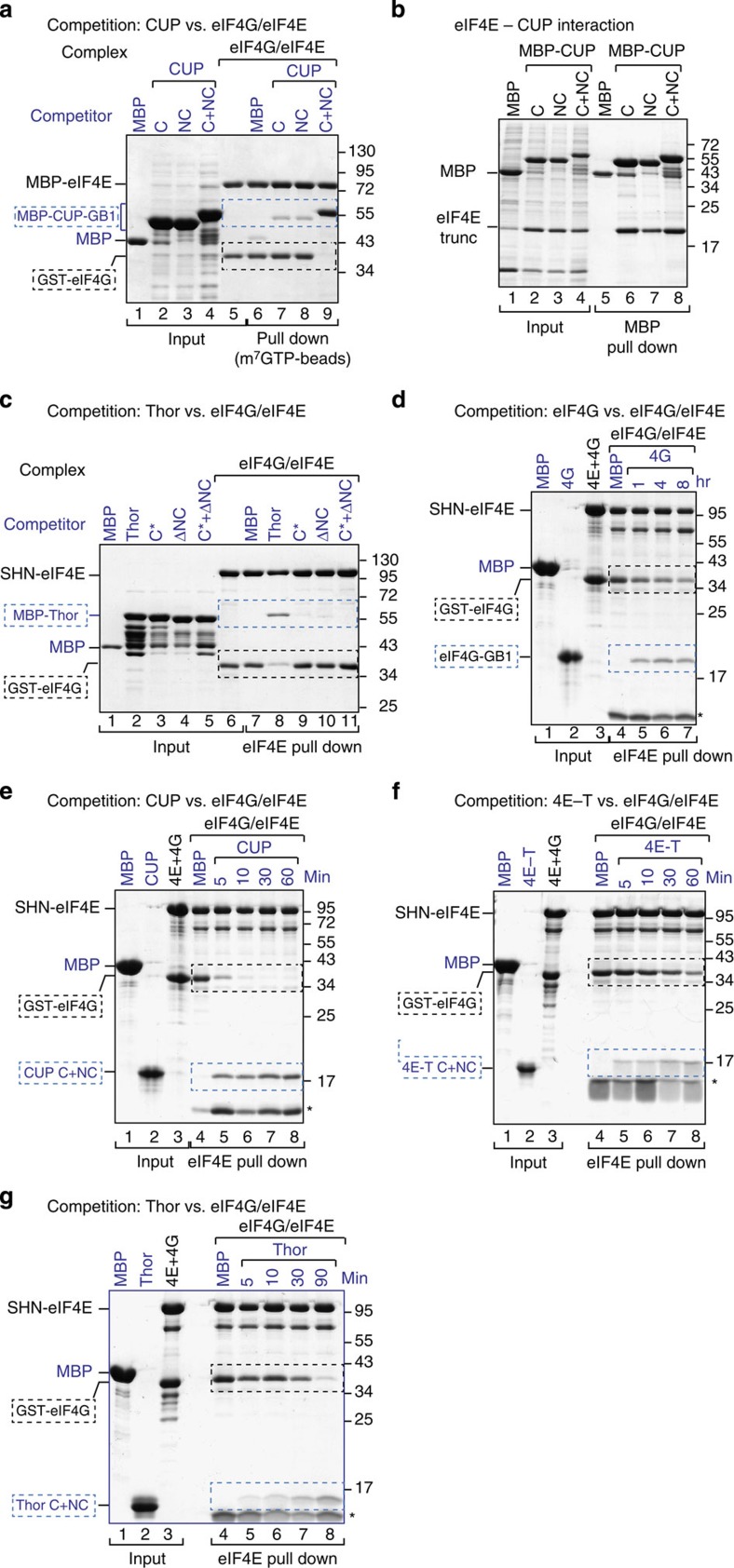
4E-BPs require the non-canonical motifs to compete with eIF4G. (**a**) Purified eIF4E–eIF4G complexes were incubated with fivefold molar excess amounts of CUP peptides containing either the C, the NC or both motifs (C+NC). The peptides were fused N terminally to MBP and C terminally to GB1. The eIF4E–eIF4G complexes contained MBP-tagged eIF4E and GST-tagged eIF4G (residues 578–650). The eIF4E-bound proteins were pulled down using m^7^GTP-sepharose beads and analyzed by SDS–PAGE. (**b**) MBP pull down showing the interaction of purified eIF4E (69–248) with the CUP fragments shown in **a**. (**c**) Purified eIF4E–eIF4G complexes were incubated with fivefold molar excess of MBP-Thor (full length, either WT or the indicated mutants). The eIF4E-bound proteins were pulled down using Strep-Tactin beads and analyzed as described in **a**. (**d**–**g**) Purified eIF4E–eIF4G complexes (1 μM) containing SHN-eIF4E (full length) and GST-eIF4G (residues 578–650) prebound to Strep-Tactin beads were incubated with a 10-fold molar excess of eIF4G (residues 578–650, **d**) and Thor (**g**) or a fivefold molar excess of CUP (**e**) and 4E-T (**f**) peptides fused C terminally to GB1. The 4E-BP peptides contained the C+NC motifs. Proteins bound to eIF4E were recovered at the indicated time points. In all of the panels, the competitor proteins are labeled in blue and highlighted by blue, dashed boxes. The black, dashed boxes mark the position of preassembled GST-eIF4G. Quantification of the dissociation assays is shown in Supplementary Fig. 7e,f. Each experiment was repeated at least twice.

Given that binding of 4E-BPs to the lateral surface of eIF4E is required for competition with eIF4G and that peptides containing only the 4E-BP C motifs cannot compete with eIF4G (Fig. 4), we next investigated the requirement for NC motifs. To this end, we performed competition assays using preassembled eIF4E–eIF4G complexes and excess 4E-BP peptides lacking either the C or NC motifs. The WT CUP C+NC peptide interacted with eIF4E and efficiently displaced preassembled eIF4G (Fig. 5a, lane 9 versus 6). Peptides containing either the C or the NC motifs of CUP did not compete with eIF4G (Fig. 5a, lanes 7 and 8), although these peptides bind to eIF4E in the absence of eIF4G (Fig. 5b, lanes 6 and 7), which is in agreement with the ITC experiments.

Similar results were obtained for Thor. Notably, deleting the non-canonical motif in the context of full-length Thor was sufficient to abolish its ability to compete with eIF4G (Fig. 5c, lane 10 versus 7), although in the absence of eIF4G this deletion mutant interacted with eIF4E (Supplementary Fig. 6a, lanes 17 and 18). Mutations in the canonical motif also abolished competition, as expected (Fig. 5c, lane 9). We conclude that 4E-BPs require both canonical and non-canonical motifs to compete with eIF4G for eIF4E binding. Thus, the non-canonical motifs play an essential role in the competition mechanism.

### 4E-BPs exhibit a kinetic competitive advantage over eIF4G

Given that the 4E-BPs and eIF4G display similar affinities for eIF4E, the differences in the ability to efficiently displace prebound eIF4G in competition assays are likely explained by the binding kinetics and the bipartite-binding mode. To obtain additional information on the ability of 4E-BPs to compete with eIF4G, we challenged preassembled eIF4E–eIF4G complexes with five- to tenfold molar excess of 4E-BP and eIF4G peptides and monitored the amount of eIF4G remaining bound to eIF4E over time.

In the absence of competitors, eIF4G remained bound to eIF4E, as expected (Fig. 5d, lane 4). In the presence of a tenfold molar excess of eIF4G peptide, we observed a 50% dissociation of prebound eIF4G after 4 h at 4 °C (Fig. 5d and Supplementary Fig. 7e). In the presence of a fivefold molar excess of CUP and 4E-T peptides (C+NC), we observed a 50% eIF4G dissociation in 2.5±0.5 and 22 min, respectively, whereas the half-life of the eIF4E–eIF4G complexes in the presence of tenfold molar excess of Thor was 37±9 min. (Fig. 5e–g, and Supplementary Fig. 7f). The simplest explanation of these results is that the bipartite-binding mode and the binding to an eIF4E surface that is not used by eIF4G confer on 4E-BPs a kinetic competitive advantage because they can bind preassembled eIF4E–eIF4G complexes without the need for prior eIF4G dissociation.

### eIF4G competes with 4E-BPs bound to the eIF4E II-AA mutant

**Figure 6:**
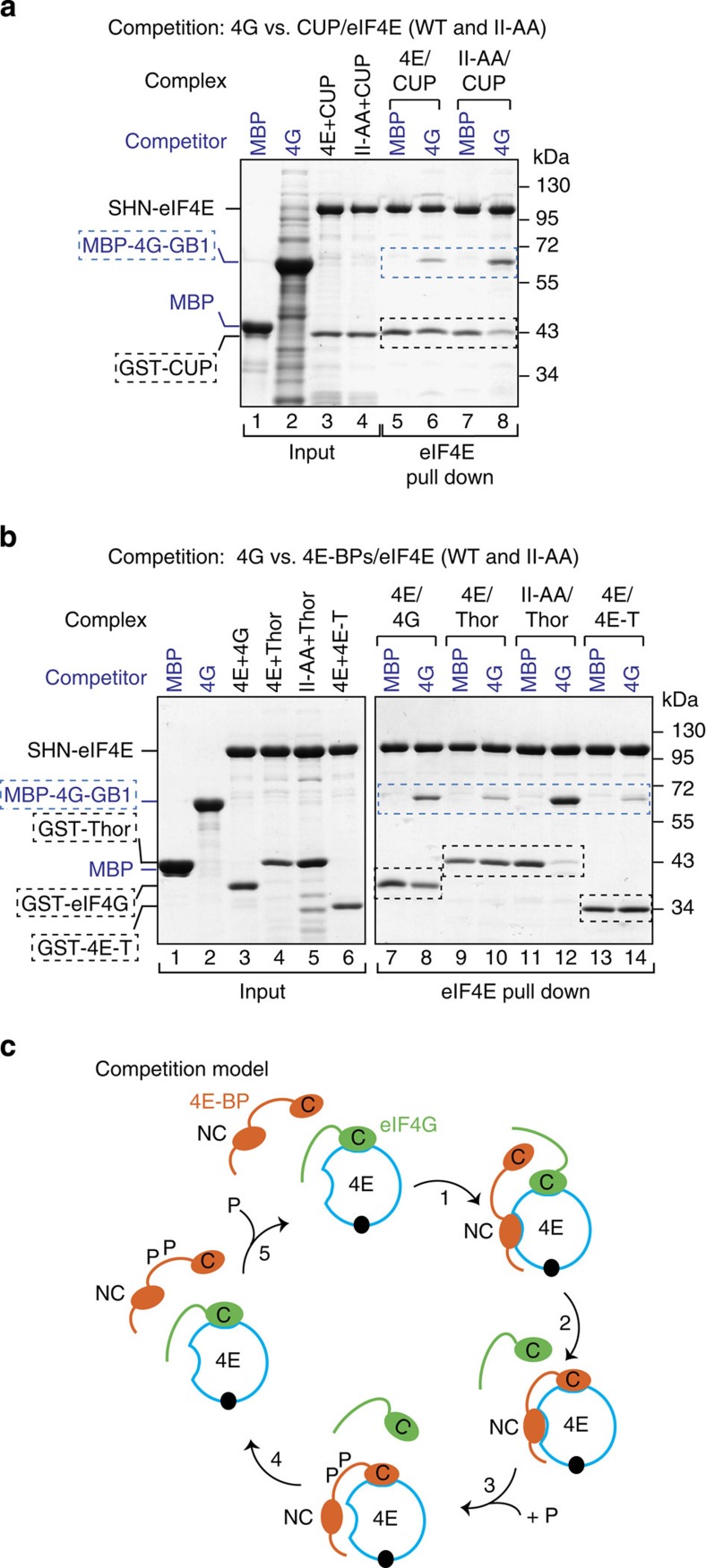
eIF4G competes with 4E-BPs when their binding to the lateral surface of eIF4E is impaired. (**a**,**b**) Purified complexes containing SHN-tagged eIF4E bound to either eIF4G or the indicated 4E-BPs were incubated with MBP or MBP-eIF4G-GB1. The amount of eIF4G or 4E-BP proteins that were associated with eIF4E was determined by pull down using Strep-Tactin beads. The complexes contained GST-eIF4G (residues 578–650), GST-CUP (residues 311–440), GST-Thor (full length) or GST-4E-T (residues 1–58). (**c**) Competition model: eIF4E (blue circle) contains a dorsal and a lateral surface that bind to the C and NC motifs of 4E-BPs (shown in orange), respectively. The dorsal surface also binds to the canonical motif of eIF4G (shown in green). The eIF4E lateral binding surface provides a docking site for the non-canonical motifs of 4E-BPs when eIF4G is bound to the dorsal surface of eIF4E via its canonical motif (1). After docking, 4E-BPs displace eIF4G from the dorsal surface of eIF4E and repress translation (2). Phosphorylation (P) of 4E-BPs destabilizes their association with eIF4E (3). Therefore, eIF4G can bind to eIF4E and translation resumes (4). In humans, 4E-BP1–3 the phosphorylation sites are located in the linker region between the 4E-BMs and in the sequences N-terminal to the canonical motif (not shown). Dephosphorylation of 4E-BPs is required for binding to eIF4E (5). Symbols are as in Fig. 3e.

Next, we asked whether eIF4G could compete with 4E-BPs when their binding to the lateral surface of eIF4E was disrupted. For this purpose, preassembled complexes containing eIF4E (WT or II-AA mutant) bound to GST-4E-BP fragments were challenged with excess amounts of MBP-eIF4G (residues 578–650). Proteins that were bound to eIF4E were recovered via eIF4E pull down. MBP-eIF4G did not displace CUP, Thor, 4E-T or eIF4G bound to WT eIF4E (Fig. 6a, lane 6 versus 5, and Fig. 6b, lanes 8, 10 and 14). In contrast, MBP-eIF4G partially displaced CUP (Fig. 6a, lane 8 versus 7) and completely displaced full-length Thor (Fig. 6b, lane 12 versus 11) bound to the eIF4E II-AA mutant. These observations indicate that eIF4G can compete with 4E-BPs for binding to eIF4E only when their interaction with the lateral surface of eIF4E is impaired. Thus, the dissociation of 4E-BPs from the lateral surface of eIF4E (for instance, on phosphorylation) may be sufficient for their dissociation from eIF4E to allow eIF4G to resume translation (Fig. 6c).

### The non-canonical motifs mediate translational repression

**Figure 7:**
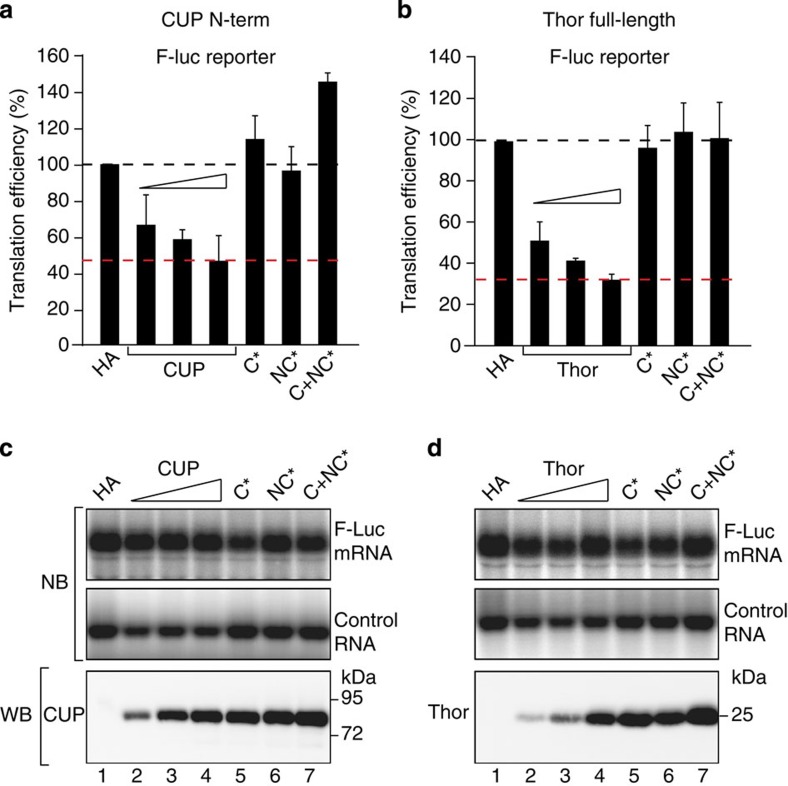
Role of non-canonical motifs in translational repression. (**a**,**b**) S2 cells were transfected with plasmids expressing a F-Luc reporter (F-Luc) and HA-CUP (N-Term, residues 1–402) or HA-Thor (full length, either WT or the indicated mutants). The F-Luc mRNA levels were analyzed by northern blotting and were normalized to those of the control RNA. The normalized F-Luc mRNA levels were used to normalize the F-Luc activity to obtain the translation efficiencies, which were set to 100 in cells expressing the HA peptide. The mean values±s.d. from three independent experiments are shown. The red, dashed lines indicate the F-Luc levels for the maximum repressive activity that was exhibited by the 4E-BPs. The black, dashed lines indicate the F-Luc levels expected in the absence of repression. (**c**,**d**) Upper panels show northern blot (NB) analyses of representative RNA samples corresponding to the experiments that are shown in **a**,**b**, respectively. The lower panels show the expression of the 4E-BP proteins analyzed by WB. The original northern and WB can be found in Supplementary Fig. 9.

To determine the role of non-canonical motifs in translational repression, we tested whether 4E-BPs repressed the expression of a firefly luciferase (F-Luc) reporter when coexpressed in S2 cells. A short uncapped and unadenylated RNA served as a transfection control (control RNA). To rule out the possibility that the inhibition of F-Luc expression resulted from changes in the F-Luc mRNA levels, we analyzed these levels by northern blotting and determined translation efficiencies (Fig. 7a,b).

The CUP N-terminal fragment or full-length Thor inhibited the expression of the F-Luc reporter in a dose-dependent manner (Fig. 7a–d). 4E-T caused mRNA degradation when overexpressed and was excluded from the analysis (C.I. and E.I., unpublished results). Mutations in either the canonical or non-canonical motifs as well as the combined mutations suppressed CUP- and Thor-mediated repression (Fig. 7a,b). The mutant proteins were expressed at levels that were comparable to the highest tested level for the WT protein (Fig. 7c,d, WB). Thus, both the canonical and non-canonical motifs are required for Thor and CUP to repress translation in a cellular context, which is in agreement with the competition assays.

### The non-canonical motifs regulate eIF4E localization

**Figure 8:**
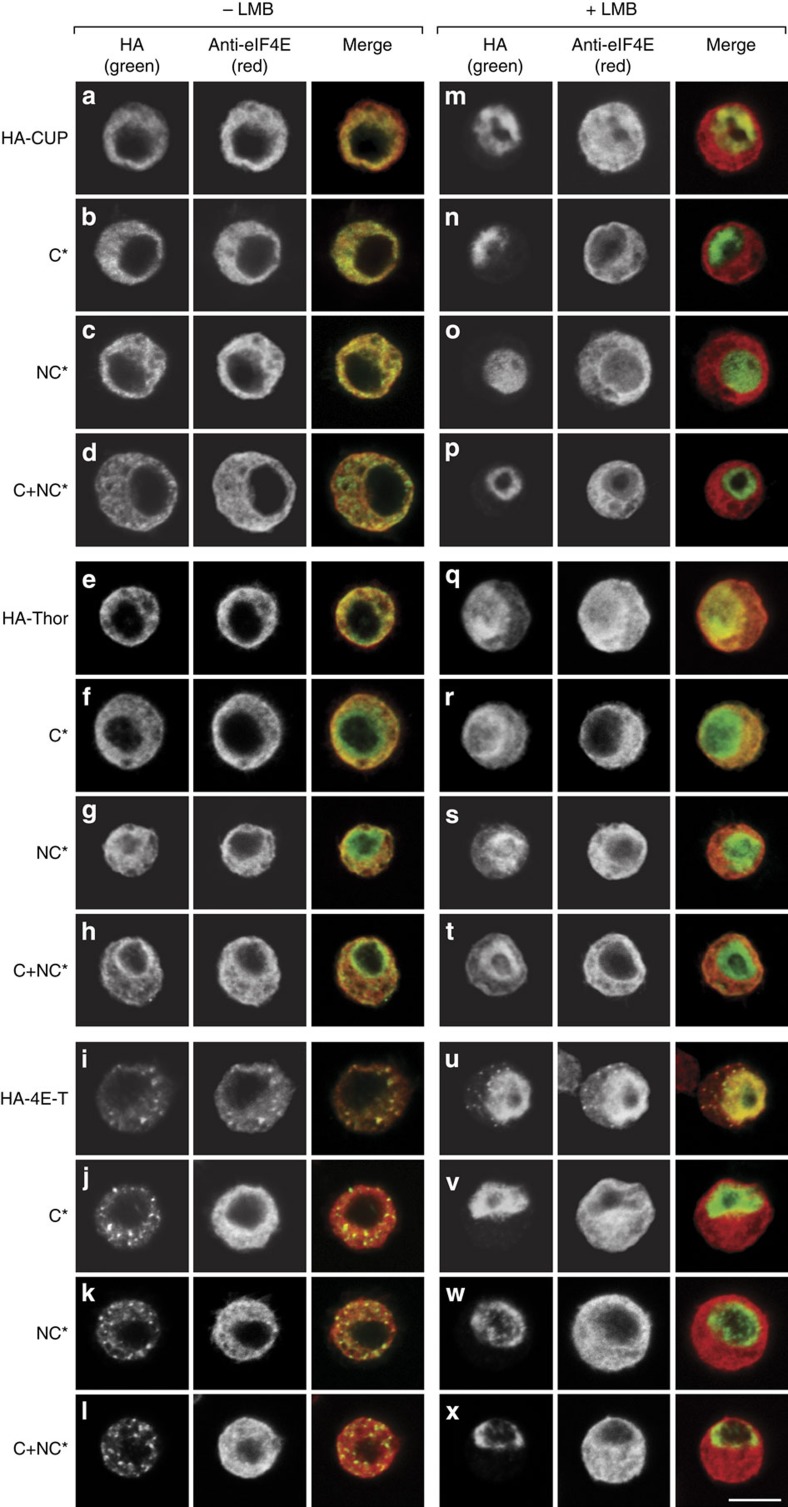
The non-canonical motifs are required for 4E-BP-mediated eIF4E nuclear import. (**a**–**x**) S2 cells expressing HA-tagged versions of CUP (**a**–**d**; **m**–**p**), Thor (**e**–**h**; **q**–**t**) or 4E-T (**i**–**l**; **u**–**x**) or the corresponding 4E-BMs mutants (indicated on the left) were treated with LMB for 12 h (+ LMB) or with methanol as control (−LMB). The cells were fixed and the localization of the HA-tagged proteins and endogenous eIF4E was determined by indirect immunofluorescence using anti-HA and anti-eIF4E antibodies. The merged pictures show the HA signal in green and the eIF4E signal in red. Scale bar, 5 μm.

4E-BPs are nucleocytoplasmic shuttling proteins that transport eIF4E to the nucleus^26^,^33^,^34^,^35^. Although eIF4E nuclear functions are not clearly understood, the nuclear retention/import of eIF4E could contribute to the efficient inhibition of cap-dependent translation. In addition, human 4E-T can also induce the accumulation of eIF4E in mRNA processing bodies or P-bodies^36^. To determine whether the non-canonical motifs contribute to the regulation of eIF4E subcellular distribution mediated by 4E-BPs, we analyzed the localization of endogenous eIF4E by immunofluorescence in S2 cells expressing WT or mutant 4E-BPs (Fig. 8).

At a steady-state, CUP and Thor distributed evenly throughout the cytoplasm (Fig. 8a,e). By contrast, 4E-T accumulated in cytoplasmic foci, which correspond to P-bodies as judged by the colocalization with the P-body marker Trailer hitch (Fig. 8i and Supplementary Fig. 7g). Endogenous eIF4E was also evenly distributed in the cytoplasm in cells overexpressing WT CUP and Thor as well as the mutant versions of these proteins (Fig. 8a–h, middle panels). In contrast, in cells expressing 4E-T, eIF4E was detected in P-bodies (Fig. 8i). Thus, 4E-T can drag eIF4E into P-bodies. Accordingly, the number of eIF4E-positive P-bodies was reduced in cells overexpressing 4E-T mutants (C*, NC*, C+NC*; Fig. 8j–l), although the mutants still localized to P-bodies. Thus, both the canonical and non-canonical motifs of 4E-T are required to induce the accumulation of eIF4E in P-bodies.

Next, we treated S2 cells with Leptomycin B (LMB), a drug that inhibits nuclear export by CRM1, which has been shown to export 4E-BPs^26^,^33^,^37^. The LMB treatment induced the nuclear accumulation of CUP and 4E-T proteins (Fig. 8m,u) and a partial nuclear accumulation of Thor (Fig. 8q). Concomitantly, endogenous eIF4E accumulated in the nucleus (Fig. 8m,q,u, middle panels). eIF4E nuclear accumulation was dependent on binding to the 4E-BPs because this accumulation was strongly reduced in cells expressing the 4E-BP mutants (Fig. 8n–p,r–t,v–x). None of the 4E-BPs required binding to eIF4E to translocate to the nucleus in the LMB-treated cells (Fig. 8m–x, left panels). Taken together, our data indicates that both the canonical and non-canonical motifs are required for 4E-BPs to regulate eIF4E subcellular distribution.

## Discussion

In this study, we show that similar to CUP, Thor and 4E-T employ a bipartite interface that is composed of canonical and non-canonical motifs to bind to the dorsal and lateral surfaces of eIF4E, respectively. While the dorsal binding surface of eIF4E is also used by eIF4G^5^,^7^,^17^, the lateral binding surface is only used by 4E-BPs and is required for 4E-BPs to displace eIF4G from preassembled eIF4E–eIF4G complexes. Based on these results, we propose that the lateral surface of eIF4E provides an exclusive docking surface for 4E-BPs on eIF4E–eIF4G complexes. After docking, 4E-BPs can begin to displace eIF4G by establishing interactions with the eIF4E dorsal surface via their own canonical motifs, further stabilizing their association with eIF4E (Fig. 6c).

The ability to bind laterally to the side of eIF4E that is not used by eIF4G enable 4E-BPs to displace eIF4G even when their binding affinities are similar and under conditions in which 4E-BPs are not in great excess compared with eIF4G. Indeed, by docking to preassembled eIF4E–eIF4G complexes, the 4E-BPs increase their local concentration and can rapidly dissociate bound eIF4G, inhibiting ongoing translation. Our model also provides one possible explanation for why eIF4G is a poor competitor compared with 4E-BPs. Indeed, eIF4G will not bind eIF4E unless the prebound eIF4G or 4E-BPs dissociate. In this context, it will be of interest to determine the contribution of the canonical and non-canonical motifs to the association (*K*_on_) and dissociation (*K*_off_) rate constants of 4E-BP proteins.

How can eIF4G bind back to eIF4E to resume translation? We show that eIF4G can displace 4E-BPs when their binding to the lateral surface of eIF4E is impaired. Although in our studies this interaction was impaired by mutations, *in vivo* this impairment could be achieved by posttranslational modifications such as phosphorylation. Indeed, it is well established that the phosphorylation of 4E-BPs reduces their affinity for eIF4E^2^,^8^. Thus, it will be of interest to dissect the impact of phosphorylation on the interaction of 4E-BPs with either the lateral or dorsal surfaces of eIF4E.

Owing to their lack of conservation, it has remained unclear whether non-canonical motifs are present in all 4E-BPs^38^. Our data indicate that the non-canonical motifs are intrinsic to the ability of 4E-BPs to compete with eIF4G and thus are likely to be present in all 4E-BPs that repress translation. At the functional level, non-canonical motifs have been proposed to play an auxiliary role and have been mainly implicated in the regulation of the affinity of eIF4E for the mRNA cap structure through allosteric effects^7^,^17^,^27^,^28^,^38^. Specifically, the binding of the 4E-BP1,2 non-canonical motifs to eIF4E increases the affinity for the cap structure^19^,^22^,^38^. Here, we show that the non-canonical motifs are essential, not auxiliary, for 4E-BP function in inhibiting translation. Given the diversity of non-canonical motifs and their different modes of interaction with eIF4E, it is possible that their binding to the lateral surface of eIF4E modulates the affinity for the cap in different ways, thereby mediating different effects. For example, by increasing the affinity of eIF4E for the cap structure, 4E-BPs may stabilize translationally repressed mRNA targets as observed for CUP^39^. Alternatively, by decreasing the affinity of eIF4E for the mRNA cap, 4E-BPs may destabilize the repressed mRNA target through decapping and subsequent decay.

In summary, our current understanding of 4E-BPs role in translational repression is predominantly based on the study of the low-molecular-weight 4E-BPs of the 4E-BP1–3 family. The identification of additional, high-molecular-weight 4E-BPs together with the characterization of their interaction mode with eIF4E reveals an unexpected sequence diversity of the eIF4E-binding regions and of the functional mechanisms. The functional diversity of 4E-BPs is further enhanced by the presence of additional domains in the high-molecular-weight 4E-BPs. These additional domains link eIF4E binding with other cellular processes, such as mRNA decay, as described for CUP and 4E-T^36^,^39^. Understanding the molecular basis for the interaction of diverse 4E-BPs with eIF4E will provide valuable insight into the variety of mechanisms that are employed by these proteins to regulate gene expression. These studies promise to uncover novel therapeutic strategies to selectively target dysregulated translation in cancer.

## Methods

### DNA constructs

The plasmids expressing the luciferase reporters, control RNA and GFP- or HA-tagged eIF4E, eIF4G, Tral and CUP (WT or mutated) have been previously described^39^,^40^,^41^,^42^. The plasmids expressing HA-Thor-V5 and GFP-Thor were obtained by inserting the corresponding DNA into the EcoRV and XhoI sites of the pAc5.1-λN-HA or pAc5.1-GFP vectors, respectively. A plasmid expressing HA-4E-T was obtained by inserting the corresponding DNA (CG32016 isoform B) into the EcoRI and NotI restriction sites of the pAc5.1-λN-HA vector. For expression in *E. coli*, the DNA encoding Thor (full length) and 4E-T (residues 1–58) was inserted into the XhoI-MfeI and AflII-NotI sites, respectively of the pnEA-NvM vector^43^ (which provides an N-terminal MBP tag followed by a TEV protease cleavage site). A DNA fragment coding eIF4G (residues 578–650) was inserted into the XhoI and BamHI restriction sites of the pnEA-NvM or pnEA-NvG (which provides an N-terminal GST tag) vector^43^.

A DNA fragment encoding full-length *Dm* eIF4E was inserted into the NdeI-BamHI restriction sites of the pnEK-NvH vector (which provides an N-terminal hexa-histidine (His_6_) tag) or the pnEK-NvSHN vector (which provides an N-terminal Strep-NusA-His-tag). The DNA fragments encoding CUP, Thor and 4E-T minimal eIF4E-binding fragments (C+NC), the individual canonical (C) and non-canonical motifs (NC, in the case of CUP), and the L+NC peptides were cloned into the NdeI-NheI restriction sites of the pnEA-NpM vector with an N-terminal MBP tag followed by an HRV3C protease cleavage site^43^. The DNA encoding the B1 domain of immunoglobulin-binding protein G (GB1)^31^ was inserted C terminally into the described fragments by site-directed insertion using the QuikChange mutagenesis kit (Stratagene). The DNA encoding a truncated eIF4E protein (residues 69–248) was cloned into the NdeI-NheI restriction sites of the pnEA-NpH vector (which provides an N-terminal His_6_-tag followed by a HRV3C protease cleavage site)^43^. All the mutants were generated by site-directed mutagenesis using the QuikChange mutagenesis kit (Stratagene) and the oligonucleotide sequences provided in Supplementary Table 3. All the constructs and mutations were confirmed by sequencing and are listed in Supplementary Table 1. The plasmids expressing full-length His_6_-eIF4E and GST-CUP (residues 311–440) were kindly provided by F. Bono^27^.

### Coimmunoprecipitation assays and western blotting

The coimmunoprecipitations assays were performed as previously described^41^. For the pull downs using m^7^GTP beads, 25 μl of immobilized γ-aminophenyl-m^7^GTP (C_10_-spacer—Jena Bioscience) beads was added to the cell lysates and the mixtures were rotated for 1 h at 4 °C. The beads were washed three times with NET buffer (50 mM Tris (pH 7.4), 150 mM NaCl, 1 mM EDTA and 0.1% Triton X-100). The bound proteins were eluted with 2 × SDS–polyacrylamide gel electrophoresis (SDS–PAGE) sample buffer and analyzed by western blotting (WB). All of the WB experiments were developed with the ECL western blotting detection system (GE Healthcare) as recommended by the manufacturer. The antibodies used in this study are listed in Supplementary Table 2.

### Protein expression and purification

Unless indicated otherwise, all the proteins were expressed in *E. coli* BL21 Star (DE3) cells (Invitrogen) that were grown in LB medium overnight at 20 °C. The lysis buffers were supplemented with DNaseI (5 μg ml^−1^), lysozyme (1 mg ml^−1^) and protease inhibitor cocktail (Roche). The truncated His_6_-eIF4E (residues 69–248) that was used in the ITC experiments and in Figs 2c and 5b was purified in lysis buffer containing 50 mM HEPES (pH 7.2), 300 mM NaCl, 20 mM imidazole and 2 mM β-mercaptoethanol using Ni^2+^-affinity chromatography (HisTrap HP 5 ml, GE Healthcare) and eluted with a gradient of 20–500 mM imidazole. After the cleavage of the His_6_-tag with HRV3C protease (homemade), the protein was further purified using a heparin column (HiTrap Heparin HP 5 ml, GE Healthcare), followed by size exclusion chromatography (Superdex 75 16/60, GE Healthcare) in 20 mM Na-phosphate (pH 7.0), 300 mM NaCl and 2 mM dithiothreitol (DTT).

To obtain the preassembled eIF4E–eIF4G complexes used in Figs 4 and 5d–g, full-length eIF4E (WT or the II-AA mutant) containing an N-terminal SNH-tag was coexpressed with an N-terminal GST-tagged eIF4G (residues 578–650). The cells were lysed by sonication in lysis buffer containing 50 mM HEPES (pH 7.2), 300 mM NaCl and 2 mM DTT. The complexes were purified from the cleared lysates using Protino Glutathione Agarose 4B beads (Machery-Nagel). The complex was further purified using a heparin column (HiTrap Heparin HP 5 ml, GE Healthcare) and a final size exclusion chromatography (Superdex 200 16/60, GE Healtcare) in 20 mM HEPES (pH 7.2), 200 mM NaCl and 2 mM DTT.

For the ITC measurements and the competition assays shown in Figs 4 and 5d–g, the 4E-BP peptides corresponding to the canonical (C) motifs, the combined (C+NC) motifs or L+NC were expressed with an HRV3C cleavable N-terminal MBP-tag and a non-cleavable C-terminal GB1 domain. The cells were lysed by sonication in lysis buffer containing 50 mM HEPES (pH 7.2), 300 mM NaCl and 2 mM DTT. The proteins were purified from the cleared lysates using amylose resin (New England Biolabs) followed by the cleavage of the MBP tag with HRV3C protease overnight at 4 °C. The proteins were further purified by size exclusion chromatography (Superdex 75 16/60, GE Healthcare) in 20 mM Na-phosphate (pH 7.0), 300 mM NaCl and 2 mM DTT. For the eIF4G fragment (residues 578–650) the MBP was removed after cleavage with TEV protease through an additional anion exchange chromatography (HiTrap Q HP 5 ml, GE Healthcare) before the final gel filtration.

### Protein pull-down assays

For the pull-down assays shown in Fig. 3 and Supplementary Fig. 6a, the full-length His_6_- or SHN-tagged eIF4E (WT or II-AA mutant) was coexpressed with GST or MBP-tagged protein fragments, including CUP (residues 311–440), Thor (full length), 4E-T (residues 1–58) or eIF4G (residues 578–650) in *E. coli* BL21 (DE3) STAR cells in autoinducing medium^44^ overnight at 20 °C. The cells were resuspended in lysis buffer (20 mM Tris–HCl (pH 7.5), 100 mM NaCl, 1 mM DTT) supplemented with EDTA-free protease inhibitor (Roche) and lysed by sonication. The cleared lysates were incubated for 1 h with 20 μl of Protino Glutathione Agarose 4B beads (Macherey-Nagel) or amylose resin (New England Biolabs). The beads were washed three times with lysis buffer, and the bound proteins were eluted with lysis buffer containing 25 mM L-glutathione or 25 mM maltose for 15 min. The proteins were analyzed by 10–15% SDS–PAGE followed by Coomassie blue staining.

In Fig. 2c, eIF4G (residues 578–650) and the C+NC peptides of CUP, Thor and 4E-T were expressed with a N-terminal MBP and a C-terminal GB1 tag. The bacterial cells were resuspended in 5 ml of lysis buffer containing 50 mM HEPES (pH 7.2), 200 mM NaCl and 2 mM DTT and lysed by sonication. Purified eIF4E (residues 69–248) was added to the cleared lysates (40–80 μl), adjusted to 0.3 ml with lysis buffer and incubated with 30 μL of amylose resin for 1 h at 4 °C. The beads were washed three times with lysis buffer and eluted with 64 μl of the same buffer containing 25 mM maltose. The proteins were analyzed by 15% SDS–PAGE followed by Coomassie blue staining.

### Competition assays

For the competition assays shown in Figs 5a,c and 6a,b, complexes containing SHN- or MBP-tagged eIF4E (WT or II-AA mutant) bound to GST-eIF4G (residues 578–650), GST-Thor (full length), GST-4E-T (residues 1–58) or GST-CUP (311–440) were obtained by coexpressing the corresponding proteins in *E. coli* BL21 (DE3) STAR cells (30 ml culture). The cells were resuspended in lysis buffer (5 ml) that was supplemented with EDTA-free protease inhibitor (Roche) and 1 mg ml^−1^ lysozyme and lysed by sonication. The cleared lysates were incubated with 400 μl of Protino Glutathione Agarose 4B (Macherey-Nagel) for 1 h. The beads were washed three times with lysis buffer, and the proteins were eluted after 10 min of incubation with lysis buffer containing 25 mM L-glutathione. The protein complexes were stored at −20 °C or used in competition assays.

The purified recombinant complexes were mixed with excess amounts of the indicated purified competitor proteins (Fig. 5c) or with bacterial lysates expressing the competitor proteins (Figs 5a and 6) and incubated for 30 min at 4 °C. After incubation, 20 μl of immobilized γ-Aminophenyl-m^7^GTP or Strep-Tactin Sepharose (IBA), were added to the samples and incubated for another 40 min at 4 °C. The beads were washed three times with lysis buffer and eluted with lysis buffer containing 2.5 mM desthiobiotin (Strep-Tactin Sepharose) or with 20 μl of SDS–PAGE loading buffer (Aminophenyl-m^7^GTP beads). The proteins were analyzed by 10–15% SDS–PAGE followed by staining with Coomassie blue staining.

For the titration experiments shown in Fig. 4, 2 μM of purified complexes containing SHN-eIF4E (WT or II-AA mutant) bound to GST-eIF4G (residues 578–650) were incubated with increasing amounts (2–20 μM) of purified competitor proteins for 20 min at 4 °C. The eIF4E-bound proteins were recovered via Strep-Tactin Sepharose pull down and eluted with lysis buffer containing 2.5 mM desthiobiotin. The proteins were analyzed by 15% SDS–PAGE followed by staining with Coomassie blue staining.

In the ‘kinetic assays’ shown in Fig. 5d–g, the purified complexes containing SHN-eIF4E and GST-eIF4G (578–650; 1 μM) were incubated with Strep-Tactin beads for 20 min. The prebound complex was then challenged with 5 μM (CUP and 4E-T) or 10 μM (Thor and eIF4G) of competitor proteins for the indicated time points. The eIF4E-associated proteins were pulled down, eluted and analyzed as described above.

### ITC analysis

The ITC experiments were performed using a VP-ITC microcalorimeter (MicroCal) at 20 °C. The solution of eIF4E (residues 69–248, WT or mutant: 1–20 μM) in the calorimetric cell was titrated with tenfold concentrated solutions of GB1-stabilized peptides corresponding to 4E-BPs C+NC (10 μM), C (50 μM), L+NC (100 μM CUP, 200 μM Thor and 4E-T) or eIF4G (residues 578–650, 20 μM) that were dissolved in the same buffer (20 mM Na-phosphate (pH 7.0) and 150 mM NaCl). The titration experiments consisted of an initial injection of 2 μl followed by 28 injections of 10 μl at an interval of 240 s. Each binding experiment was repeated twice. The thermodynamic parameters were estimated using a one-site binding model (Origin version 7.0), whereby the datapoint of the first injection was removed for the analysis^45^.

### Translation repression assays

S2 cells were transfected in 6-well plates using Effectene transfection reagent (Qiagen) according to the manufacturers protocol. The transfection mixtures contained: 0.1 μg of F-Luc reporter plasmid (F-Luc-V5), 0.3 μg of control RNA reporter, and increasing amounts of plasmids expressing HA-CUP (fragment 1–402; 0.05–0.2 μg) and HA-Thor (full length, 0.1–0.5 μg). The plasmids expressing the corresponding mutants or the HA peptide control were transfected at the highest concentration. In all the experiments, the cells were collected three days after transfection. The F-Luc activity was measured using the Dual-Luciferase reporter assay system (Promega). The northern blotting was performed as previously described^42^. The F-Luc mRNA levels were determined by northern blotting and were normalized to those of the control RNA. The normalized F-Luc mRNA levels were then used to normalize the F-Luc activity, to obtain translation efficiencies.

### Immunofluorescence

S2 cells expressing HA-tagged versions of CUP, Thor and 4E-T or the indicated mutants were treated with Leptomycin B (100 nM; Sigma) or methanol as a control for 12 h. After the LMB treatment, the cells were allowed to adhere to poly-D-lysine–coated coverslips for 15 min and were fixed with 2% paraformaldehyde for 10 min. The cells were then permeabilized with 0.1% Triton X-100 in PBS (10 min) and stained with affinity-purified monoclonal anti-HA (Covance 1:1,000) and polyclonal anti-eIF4E (1:2,000) antibodies in PBS containing 1% BSA for 1 h. Alexa Fluor 594-labeled goat anti-rabbit and 488-labeled anti-mouse antibodies (Invitrogen) were used at dilutions of 1:1,000 and 1:2,000, respectively. The cells were mounted using Fluoromount-G (Southern Biotech). The images were acquired at room temperature using a confocal microscope (TCS SP2; Leica) that was fitted with a Plan-Apochromat × 100 NA 1.40 oil immersion objective and a series of three photomultipliers (Hamamatsu Photonics) controlled with the Leica confocal software (version 2.61). The images were prepared using Photoshop (Adobe).

## Author contributions

C.I. performed most of the experiments and supervised assays performed by C.W.; D.P. contributed to some experiments, performed ITC measurements and contributed to the writing of the manuscript; C.W. performed pull-down assays and prepared several DNA constructs; E.I. was the principal investigator who coordinated the project; C.I. and E.I. designed the research project and drafted the manuscript.

## Additional information

**How to cite this article**: Igreja, C. *et al.* 4E-BPs require non-canonical 4E-binding motifs and a lateral surface of eIF4E to repress translation. *Nat. Commun.* 5:4790 doi: 10.1038/ncomms5790 (2014).

## Supplementary Material

Supplementary Information Supplementary Figures 1-9 and Supplementary Tables 1-3

## Figures and Tables

**Table 1:** Thermodynamic parameters for the interaction of eIF4E with eIF4G and 4E-BP peptides.

	**K**_**D**_ **(M)**	**ΔH (kcal mol**^**−1**^**)**	**TΔS (kcal mol**^**−1**^**)**	**ΔG (kcal mol**^**−1**^**)**	**Molar ratio**
*Peptide+eIF4E*					
eIF4G 578-680	17±13 × 10^−9^	−18.5±2	7.97	−10.51	0.99±0.08
CUP C+NC	9.1±0.5 × 10^−9^	−34.5±0.35	23.86	−10.78	1.05±0.02
CUP C	1.6±0.1 × 10^−7^	−16.84±0.04	7.70	−9.13	1.01±0.01
CUP L+NC	1.03±0.03 × 10^−7^	−18.5±0.2	9.10	−9.38	0.98±0.01
Thor C+NC	1.4±0.3 × 10^−9^	−16.8±1	4.90	−11.87	0.95±0.02
Thor C	2.26±0.06 × 10^−6^	−12.1±2.8	2.24	−9.82	1.06±0.01
Thor L+NC	nb	nb	nb	nb	nb
4E-T C+NC	5.6±2.4 × 10^−9^	−22.8±3	11.73	−11.11	0.95±0.01
4E-T C	1.6±0.2 × 10^−8^	−18.6±0.7	8.16	−10.45	0.95±0.01
4E-T L+NC	nb	nb	nb	nb	nb
					
*Peptide+eIF4E (II-AA)*					
eIF4G 578-680	40±9.5 × 10^−9^	−16.24±0.04	6.32	−9.93	1.03±0.01
CUP C+NC	5.0±0.8 × 10^−8^	−18.6±0.7	8.79	−9.84	0.98±0.01
Thor C+NC	4.7±0.3 × 10^−7^	−7.6±0.3	−0.91	−8.46	0.97±0.01
4E-T C+NC	8.8±2 × 10^−9^	−12.9±0.3	2.16	−10.80	1.03±0.04

C, canonical; eIF4, eukaryotic initiation factor 4; L, linker; nb, no binding; NC, non-canonical.

See Supplementary Figs 3–5.
