# Supplementary material for: 4E-BPs require non-canonical 4E-binding motifs and a lateral surface of eIF4E to repress translation
Source: Nat Commun. 2014 Sep 2;5:4790. doi: 10.1038/ncomms5790 (PMC4164784; doi:10.1038/ncomms5790)
Supplement: Supplementary Information — Supplementary Figures 1-9 and Supplementary Tables 1-3 [file ncomms5790-s1.pdf]

Supplementary Figures

Supplementary Fig. 1

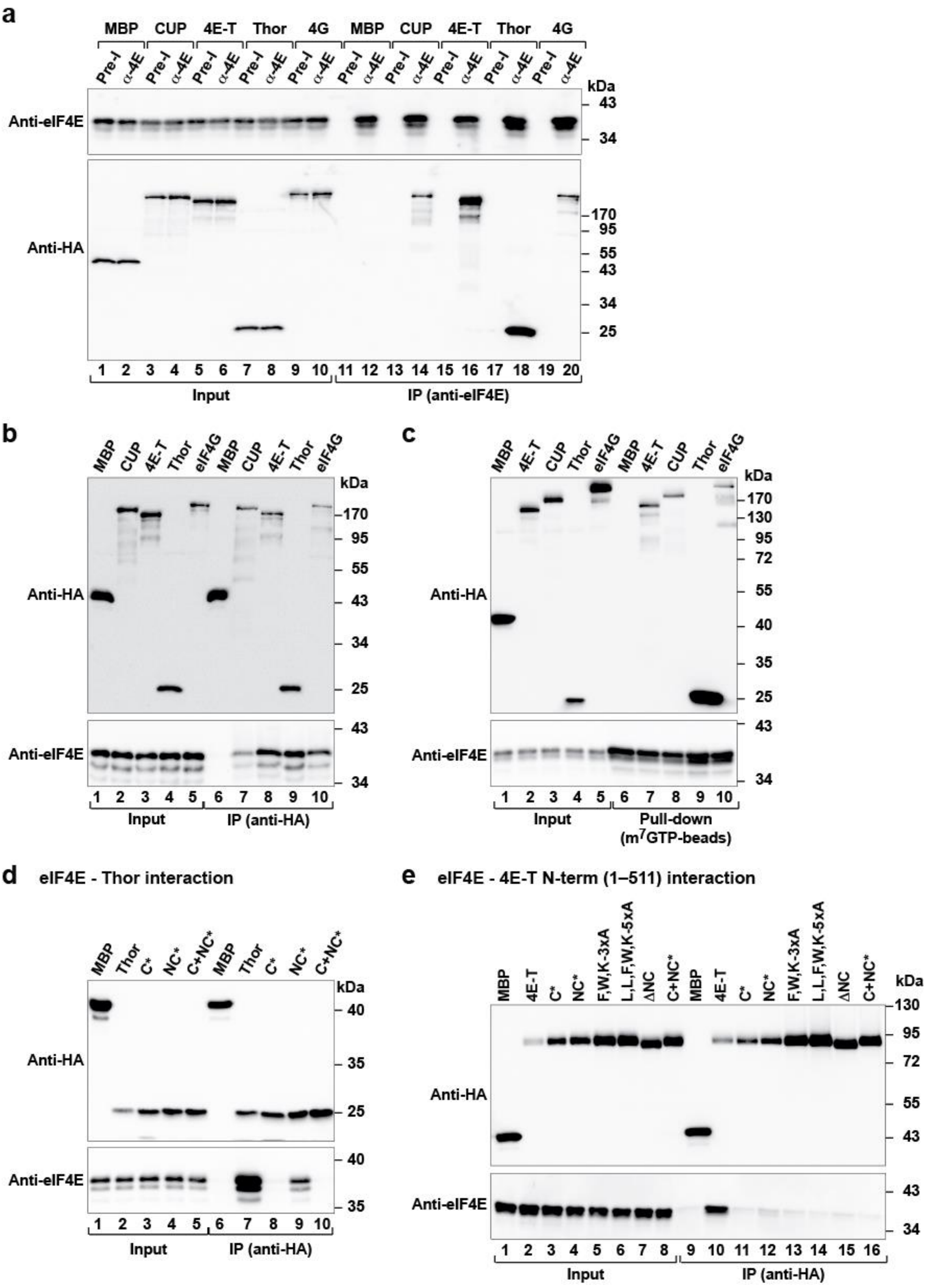

Supplementary Figure 1. The interaction of eIF4E with 4E-BPs and eIF4G in S2 cell lysates. (**a**) Western blot showing the interaction of HA-tagged full length 4E-BPs (CUP,

Thor and 4E-T) or HA-eIF4G and endogenous eIF4E in S2 cell lysates. The proteins were immunoprecipitated using a polyclonal anti-eIF4E antibody (α-4E). The pre-immune (Pre-I) serum and an HA-tagged version of MBP served as negative controls. The inputs (1%) and immunoprecipitates (10%) were analyzed by western blotting using anti-HA and anti-eIF4E antibodies. (**b,c**) The interaction of HA-tagged full length 4E-BPs (CUP, Thor and 4E-T) and eIF4G with endogenous eIF4E was analyzed by coimmunoprecipitation using anti-HA antibodies (**b**) or by pull-down using m^7GTP-Sepharose beads (**c**). HA-MBP served as a negative control. The inputs (10%) and immunoprecipitates (30%) were analyzed by western blotting using anti-HA and anti-eIF4E antibodies. (**d**) Western blot showing the interaction of HA-Thor (full-length either wild-type or mutants) with endogenous eIF4E in S2 cells. The proteins were immunoprecipitated using anti-HA antibodies and analyzed as described in (**b**,**c**). (**e**) Western blot showing the interaction of HA-4E-T (residues 1–511, wild-type or mutants) with endogenous eIF4E. The proteins were immunoprecipitated using anti-HA antibodies and analyzed as described in (**b**,**c**). The size markers (kDa) are shown to the right of each panel. Mutants are described in Supplementary Table 1.

Supplementary Fig. 2

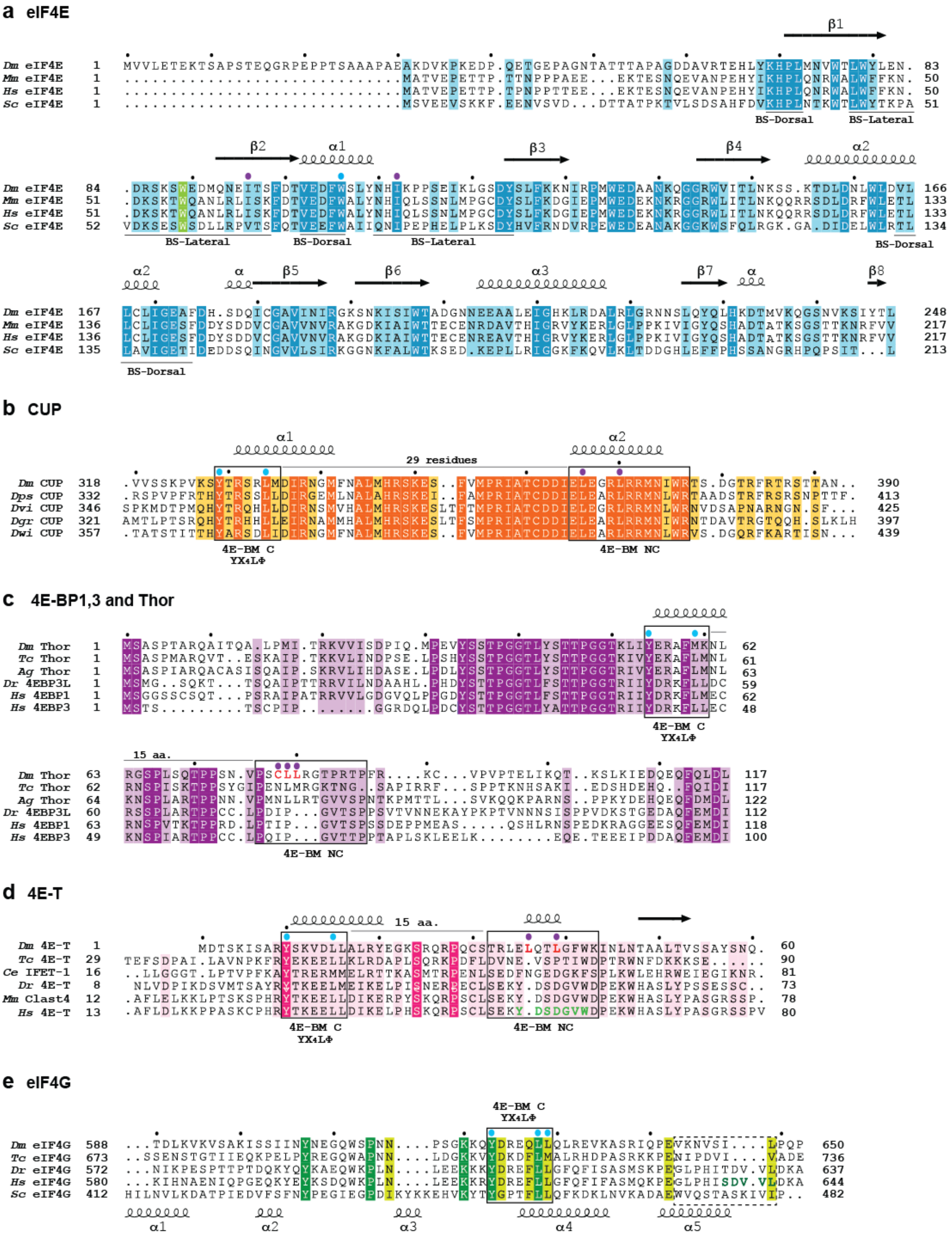

Supplementary Figure 2. Sequence alignments. Residues conserved in all aligned sequences are highlighted with a dark color background and printed in white. Residues with >70% similarity are shown with a light color background. (**a**) Structure-based sequence

alignment of eIF4E proteins from *Drosophila melanogaster* (*Dm*), *Mus musculus* (*Mm*), *Homo sapiens* (*Hs*) and *Saccharomyces cerevisae* (*Sc*). Secondary structure elements of the *Dm* eIF4E (PDB code: 4AXG)[27] are indicated above the sequences. The lateral and dorsal binding surfaces (BS) are indicated with a line below the sequences. Residues mutated in this study are indicated by filled circles above the sequence. Circles are colored in cyan or magenta for the dorsal and lateral surfaces, respectively. (**b**) Sequence alignment of the eIF4E-interacting regions of CUP orthologous proteins from insects. Species are as follows: *Drosophila melanogaster* (*Dm*), *Drosophila pseudoobscura* (*Dps*), *Drosophila virilis* (*Dvi*), *Drosophila grimshawi* (*Dgr*), *Drosophila willistoni* (*Dwi*). Secondary structure elements of the *Dm* CUP (PDB code: 4AXG) protein are indicated above the sequences. The canonical (C) and non-canonical (NC) 4E-BMs are boxed in black. Residues mutated in this study are indicated with filled circles above the sequence. Circles are colored in cyan or magenta for the canonical and non-canonical 4E-BMs, respectively. (**c**) Sequence alignment of Thor orthologous proteins. Species are as follows: *Drosophila melanogaster* (*Dm*), *Tribolium castaneum* (*Tc*), *Anopheles gambiae* (*Ag*), *Danio rerio* (*Dr*) and *Homo sapiens* (*Hs*). Additional symbols are as described in panel (**b**). (**d**) Alignment of the eIF4E-interacting region of 4E-T orthologous proteins. Species are *Caenorhabditis elegans* (*Ce*) and as in (a,**c**). Predicted secondary structure elements are indicated above the sequences. A putative non-canonical 4E-BM identified in the *Hs* 4E-T protein is highlighted in green characters. Additional symbols are as described in panel (**b**). (**e**) Alignment of eIF4E-interacting regions of eIF4G orthologous proteins. Species are as in panels (**a,d**). A putative non-canonical 4E-BM identified in the *Hs* eIF4G protein and the corresponding motif in *Dm* are indicated by a dashed box. Secondary structure elements of the *Sc* eIF4G (PDB 1RF8) protein are indicated below the sequences. Additional symbols are as described in panel (**b**).

Supplementary Fig. 3

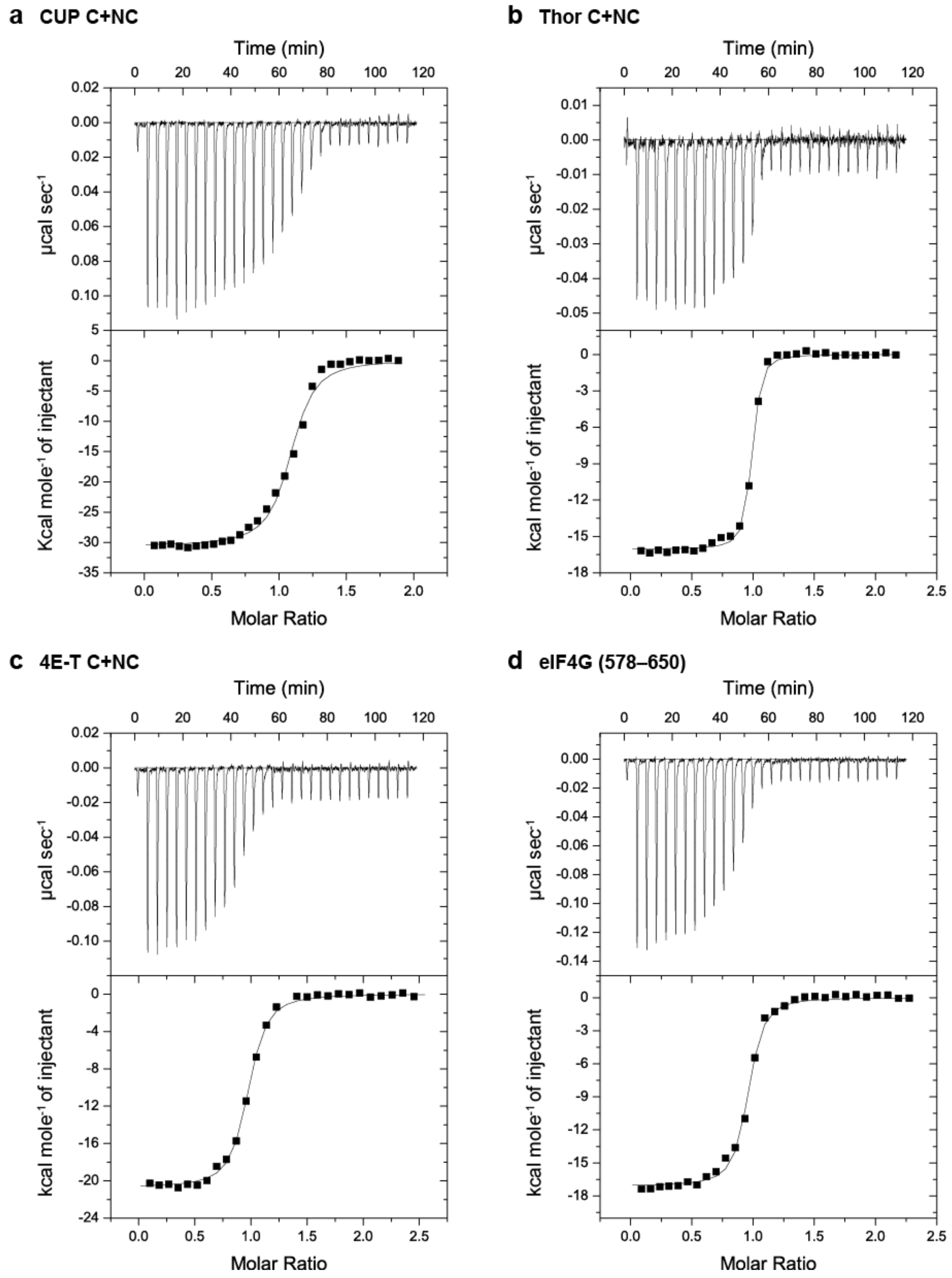

Supplementary Figure 3. Thermodynamic parameters for the interaction of eIF4E with eIF4G and 4E-BP peptides. (**a–d**) ITC profiles of CUP (**a**), Thor (**b**), 4E-T (**c**) and eIF4G

(**d**) minimal regions for binding to eIF4E (wild-type (WT), residues 69–248). The top panels represent the raw data (μcal sec^{-1}) whereas the bottom panels show the integrated data (kcal $mole^{-1}$ of injectant) of heat changes that were best-fitted using a one-site binding model. The thermodynamic parameters correspond to heat changes that were detected when 4E-BPs (10 μM) or eIF4G (20 μM) were injected into the calorimetric cell (1.4 mL) containing 1 μM (**a,b,c**) or 2 μM (**d**) eIF4E.

Supplementary Fig. 4

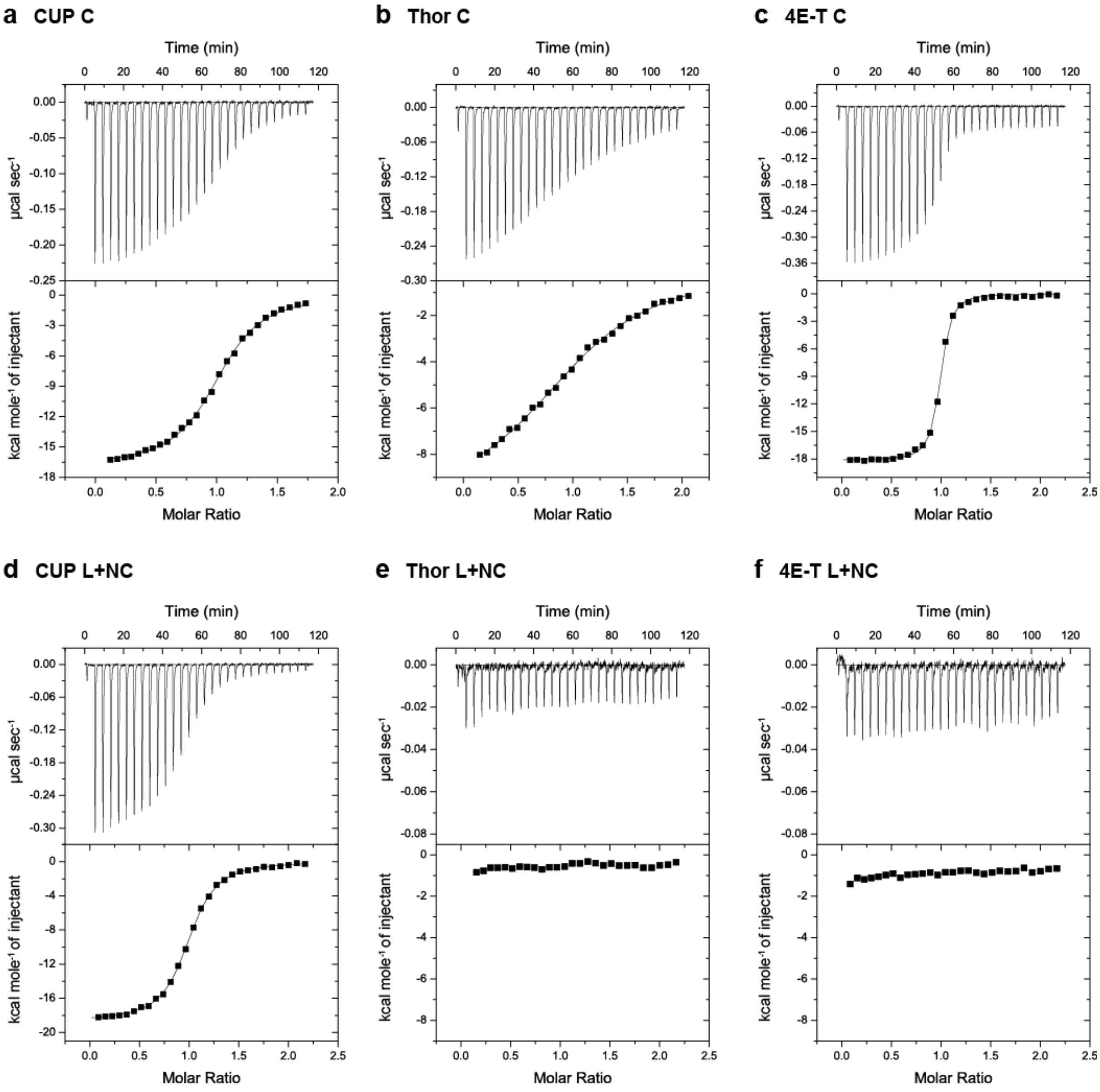

Supplementary Figure 4. Thermodynamic parameters for the interaction of eIF4E with 4E-BP peptides. (**a–f**) ITC profiles for the interaction of eIF4E (WT, residues 69-248) and CUP (**a,d**), Thor (**b,e**) and 4E-T (**c,f**) peptides containing only the canonical motifs (**a–c**) or the L+NC regions (**d–f**). The measurements were performed as described in Supplementary Fig. 3. The solutions contained the peptides corresponding to the canonical motifs (50 µM) or the L+NC regions (100 µM for CUP and 200 µM for Thor and 4E-T). The eIF4E concentration in the calorimetric cell was 5 µM (for the C peptides), 10 µM (for CUP L+NC) and 20 µM (for Thor and 4E-T L+NC peptides).

Supplementary Fig. 5

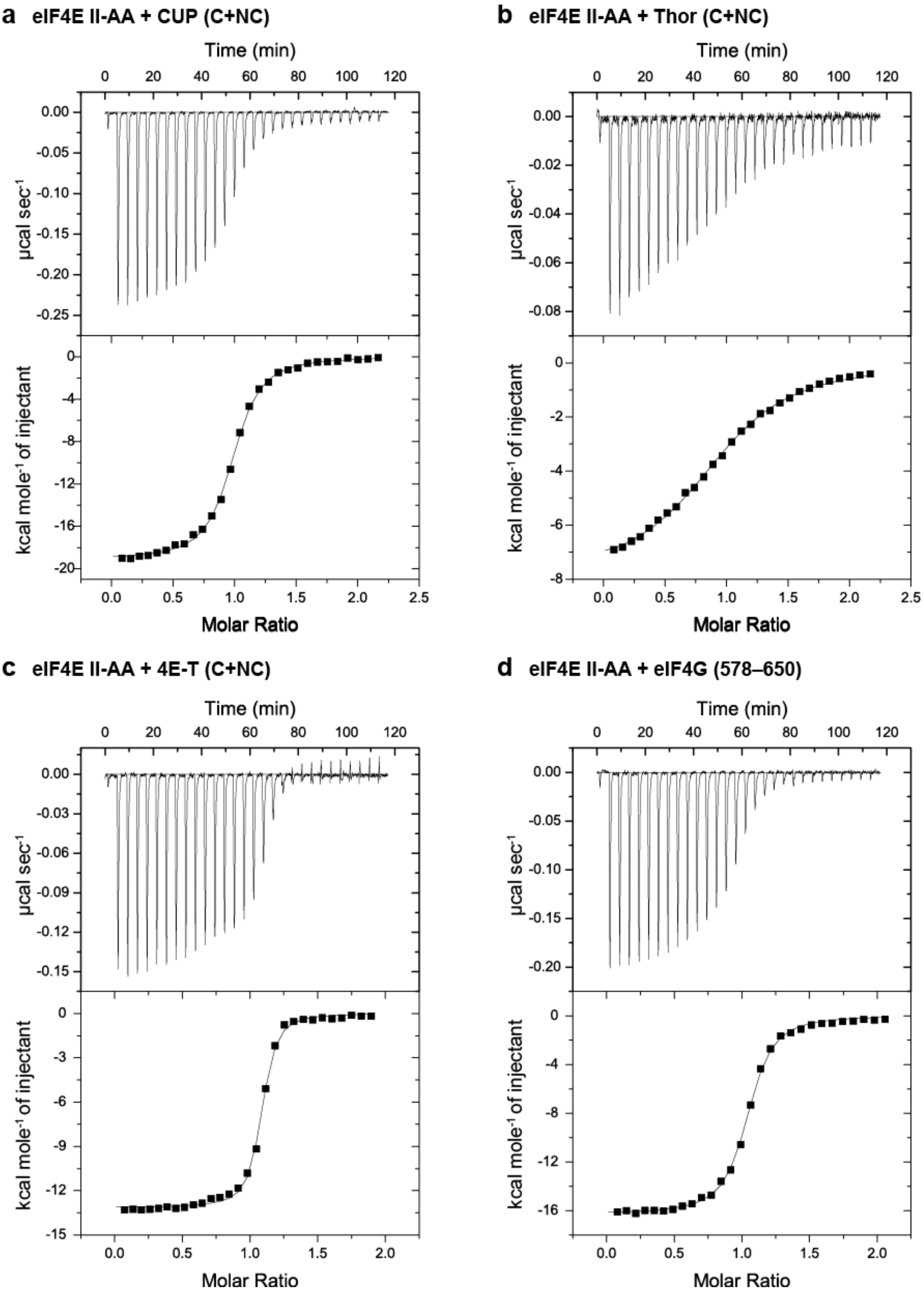

Supplementary Figure 5. Thermodynamic parameters for the interaction of eIF4E II-AA mutant with 4E-BP peptides. (**a–d**) ITC profiles for the interaction of eIF4E IIAA

mutant (residues 69-248) and CUP (**a**), Thor (**b**), 4E-T (**c**) and eIF4G (**d**) peptides. The measurements were performed as described in **Supplementary Fig. 3**. The solutions contained the 4E-BP C+NC peptides (10 µM) and 20 µM for the eIF4G peptide. The eIF4E II-AA mutant concentration in the calorimetric cell was 5 µM.

Supplementary Fig. 6

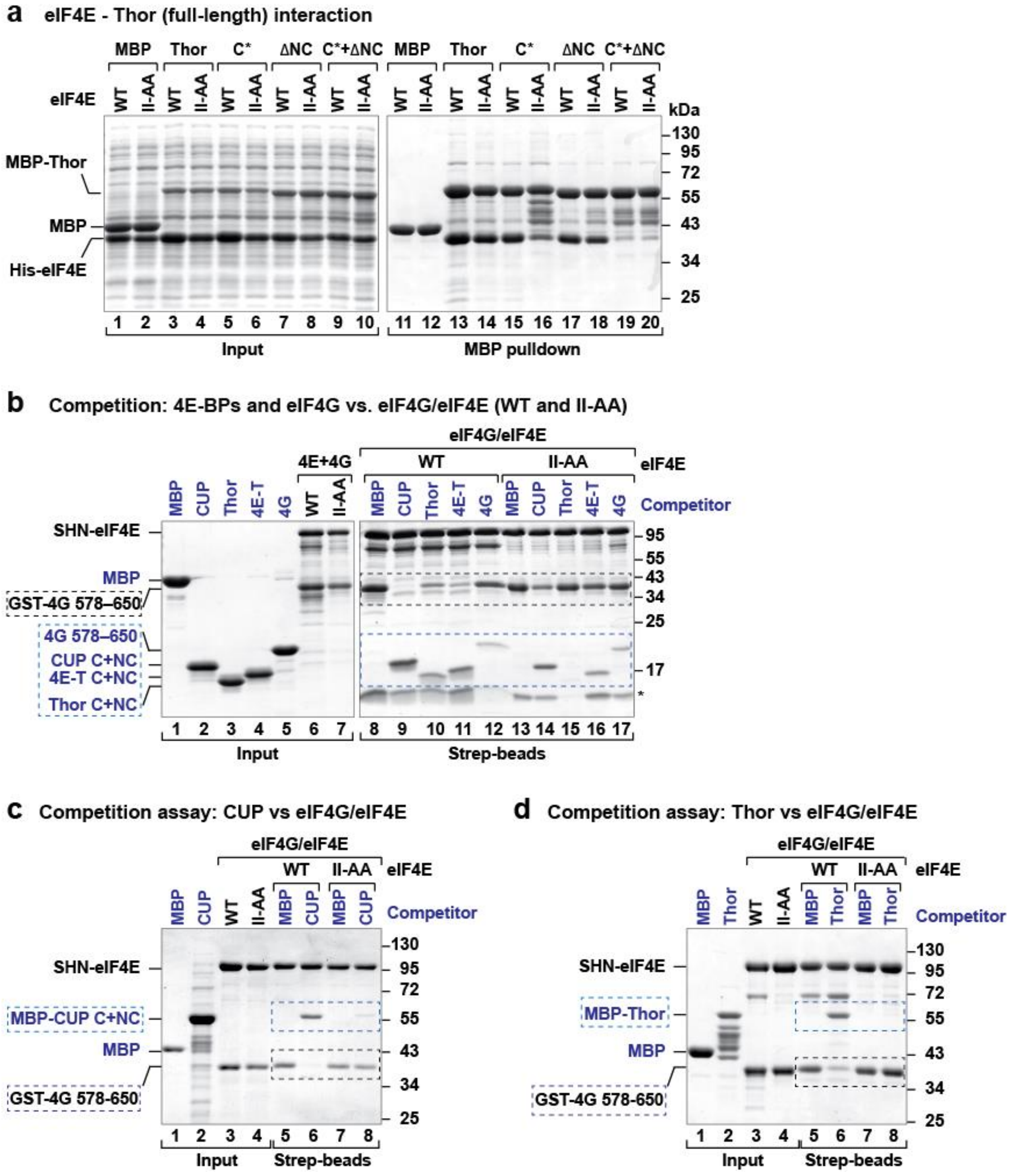

Supplementary Figure 6. Identification of a non-canonical 4E-BM in Thor and competition assays. (**a**) The association of MBP-Thor (full length, wild-type or 4E-BM mutant) with His_6-eIF4E (WT or II-AA mutant) was analyzed by MBP pull-down as described in Fig. 3c. (**b**) Purified eIF4E–eIF4G complexes containing SHN-eIF4E (wild-type or II-AA mutant) and GST-eIF4G (578–650) were incubated with CUP, Thor and 4E-T fragments (C+NC) fused C-terminally to GB1. The proteins that were bound to eIF4E were

pulled down using Strep-Tactin beads and analyzed by SDS-PAGE. The competitor peptides are labeled in blue, and their positions are highlighted by blue, dashed boxes. The black, dashed boxes indicate the position of preassembled GST-eIF4G. (**c,d**) The purified eIF4E–eIF4G complexes described in panel (**b**) were incubated with a 5-fold molar excess of CUP (**c**) (C+NC fragment) or MBP-Thor full length (**d**). The eIF4E-bound proteins were pulled down using Strep-Tactin beads and analyzed by SDS-PAGE. The size markers (kDa) are shown to the right of each panel.

Supplementary Fig. 7

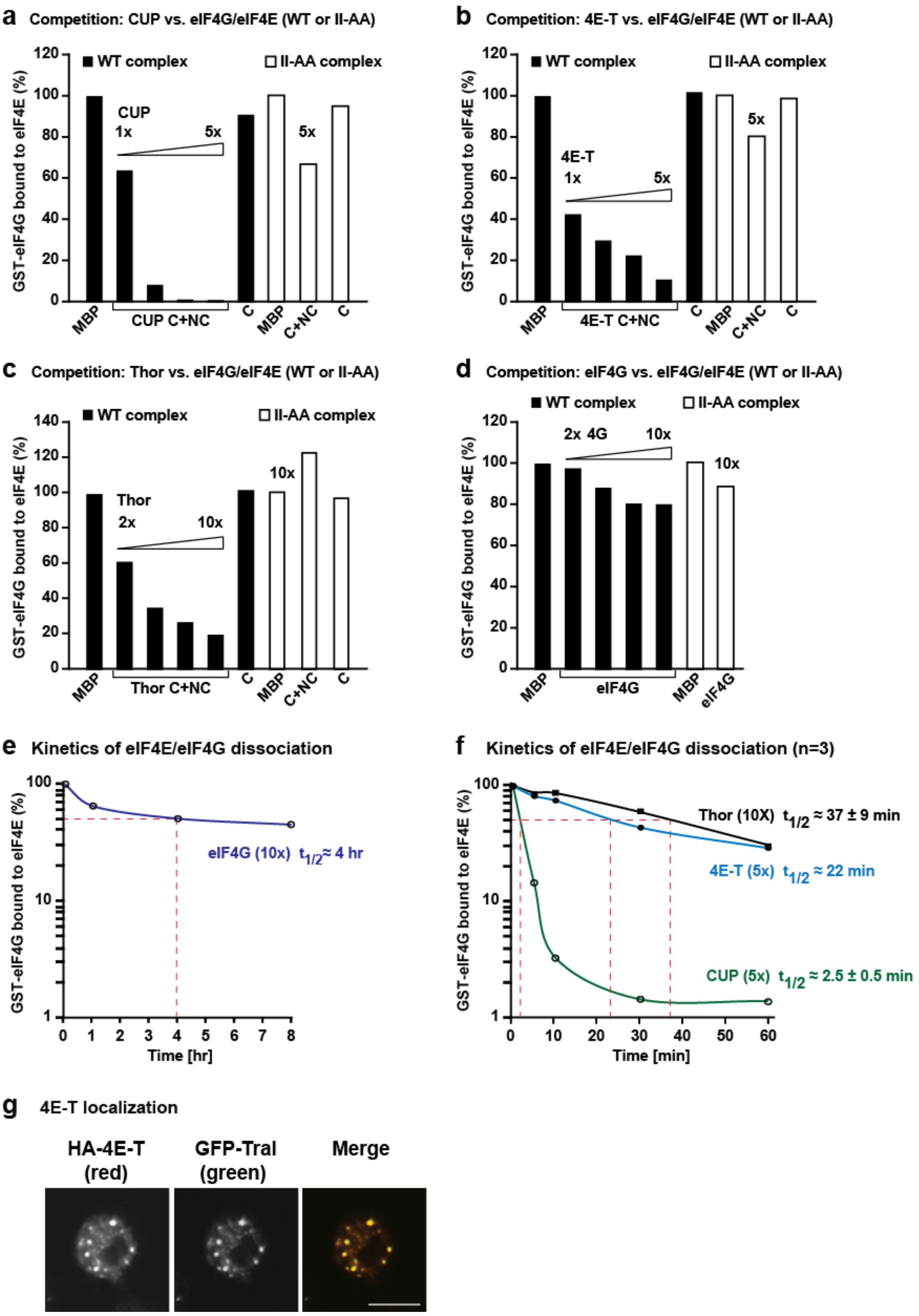

Supplementary Figure 7. Quantitative analysis of the competition assays. (a–d) Quantification of GST-eIF4G remaining bound to eIF4E (WT or II-AA mutant) in the

presence of MBP (negative control), increasing amounts of 4E-BP peptides or eIF4G-GB1 in the experiments shown in Fig. 4a–d. The amounts of GST-eIF4G bound to eIF4E in each experimental condition were determined using the Image J software after Coomassie blue staining. To rule out the possibility that changes in the amount of GST-eIF4G bound to eIF4E resulted from variations in the loading volume, all values were normalized to the levels of SHN-eIF4E present in each condition. These values were set to 100 in the presence of MBP. Each experiment was repeated at least twice. (**e,f**) Quantification of the experiments shown in Fig. 5d–g. The amounts of GST-eIF4G remaining bound to eIF4E in the presence of MBP, 4E-BP peptides or eIF4G-GB1 were normalized to those of eIF4E and set to 100 in the presence of MBP. The data represent averages of two (eIF4G and 4E-T) or three (CUP and Thor) independent experiments. (**g**) *Dm* 4E-T localizes to P-bodies. Confocal fluorescent micrographs of fixed S2 cells expressing HA-4E-T and GFP-Tral. The localization of HA-4E-T was determined by indirect immunofluorescence using an anti-HA antibody. The merged pictures show the HA signal in red and the GFP-Tral signal in green. Bar: 5 µm.

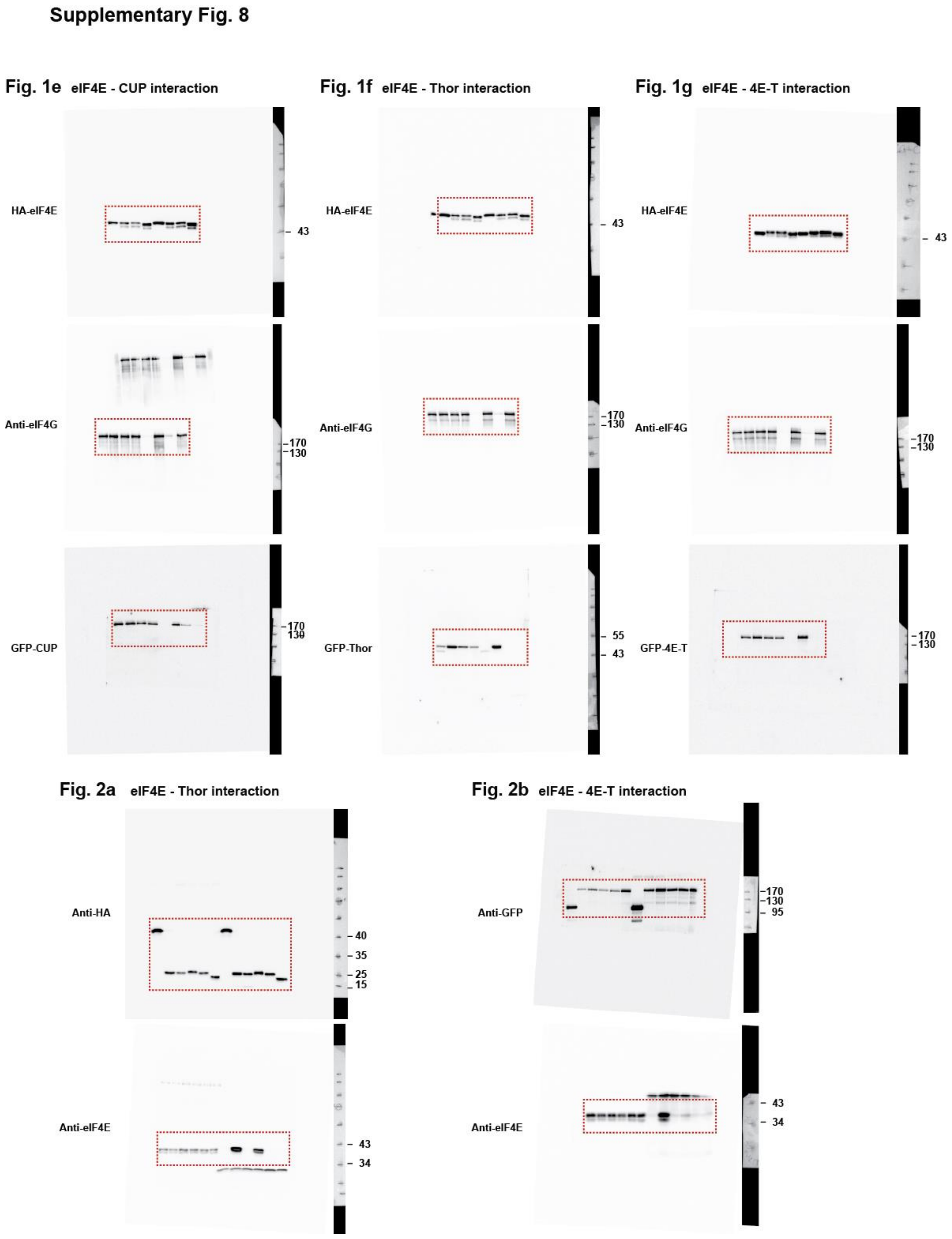

Supplementary Figure 8. Original images of western blots used in Fig. 1e–g and Fig. 2a,b.

Supplementary Fig. 9

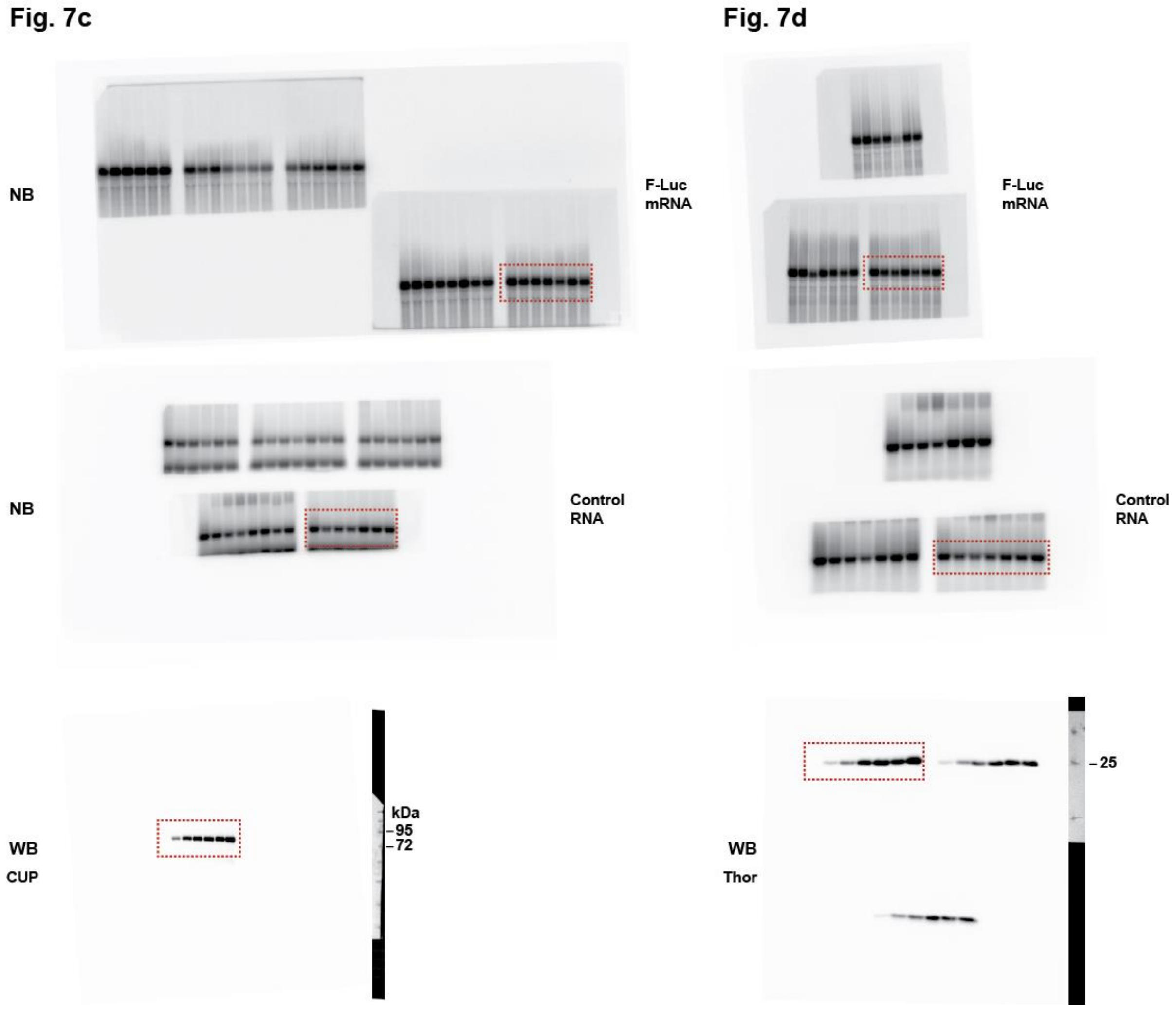

Supplementary Figure 9. Original images of western and northern blots used in Fig. 7c,d.

Supplementary Table 1. Mutants and constructs used in this study

Protein	Name of the construct	Mutations / fragments	Binding site / motif
**eIF4E** (1–248) (isoform C) P48598-2	W106A	W106A	Dorsal surface
	II-AA	I96A, I112A	Lateral surface
	trunc	69–248	
**eIF4G** (1–1666) (isoform A) O61380	4G	578–650	eIF4E-binding region
	C*	Y621A, L626A, L627A	Canonical
**CUP** (1–1117) Q9VMA3	CUP	311–440	eIF4E-binding region
	C+NC	325–376	eIF4E-binding region short
	C	325–341	Canonical
	NC	362–376	Non-canonical
	L+NC	342–376	Linker+non-canonical
	C*	Y327A, L332A	Canonical
	NC*	L364A, L368A	Non-canonical
	C+NC*	Y327A, L332A, L364A, L368A	Double mutant
	N-term	1–402	N-terminus
**Thor** (1–117) Q9XZ56	Thor	Full length	
	C+NC	50–83	eIF4E-binding region short
	C	50–63	Canonical
	NC	77–83	Non-canonical
	L+NC	64–83	Linker+non-canonical
	C*	Y54A, M59A	Canonical
	NC*	C78A, L79A, L80A	Non-canonical
	R,G,T	R81A, G82A, T83A	Non-canonical
	ΔNC	Δ76–84	Non-canonical
	C+NC*	Y54A, M59A, C78A, L79A, L80A	Double mutant
	C*+ΔNC	Y54A, M59A, Δ76-85	Double mutant
**4E-T** (1–1010) Q8IH18	4E-T	1–58	eIF4E-binding region
	C+NC	9–44	eIF4E-binding region short
	C	9–21	Canonical
	NC	31–44	Non-canonical
	L+NC	22–44	Linker+non-canonical
	C*	Y10A, L15A	Canonical
	NC*	L36A, L39A	Non-canonical
	F,W,K-3xA	F41A, W42A, K43A	Non-canonical
	L,L,F,W,K-5xA	L36A, L39A, F41A, W42A, K43A	Non-canonical
	ΔNC	Δ28–43	Non-canonical
	C+NC*	Y10A, L15A, L36A, L39A	Double mutant
	C*+ΔNC	Y10A, L15A, Δ28-43	Double mutant
	N-term	1–511	N-terminus

Supplementary Table 2. Antibodies used in this study

Antibody	Source	Catalog Number	Dilution	Monoclonal/ Polyclonal
Anti-HA-HRP (for Western blot)	Roche	12 013 819 001	1:5,000	Monoclonal
Anti-eIF4E	In house		1:3,000	Rabbit polyclonal
Anti-GFP	In house		1:2,000	Rabbit polyclonal
Anti-eIF4G	In house		1:3,000	Rabbit polyclonal
Anti-HA (for Immunofluorescence)	Covance	MMS-101P	1:1,000	Monoclonal
Anti-rabbit-HRP	GE Healthcare	NA934V	1:10,000	Polyclonal
Alexa Fluor 594–labelled goat anti–rabbit	Invitrogen	A-11080	1:1,000	Polyclonal

Supplementary Table 3. Mutagenesis primers used in this study

Protein	Mutations	Primer sequence 5'- 3'(Forward)
eIF4E	W106A	cttcgataccgtcgaggacttcgcgagcctatacaaccacatcaagccc
	II-AA	1 - gggaggacatgcaaaacgaggccaccagcttcgataccgtcga<br>2 - tctggagcctatacaaccacgccaagcccccatcagagatcaa
eIF4G	Y621A, L626A, L627A	cggaaaaaaacaagctgaccgagaacaggctgctcagttacgcga
CUP	Y327A, L332A	aagccggtcaagagcgctacccgctcccgcgcgatggacattcgcaac
	L364A, L368A	ccacctgcgacgacattgaggcggagggaagggctcgccgcatgaatatttggcgc
Thor	Y54A, M59A	ctcctggaggcaccaaacttatcgccgagcgggctttcgcgaagaatctccgtggctccccatt
	C78A, L79A, L80A	ctccgccgtccaacgtgcccagtgctgcggcgaggggcactccgcgtactccctt
	R81A, G82A, T83A	ccaacgtgcccagttgcttgctggctgccgctccgcgtactcccttccgcaagtg
	Δ76–84	gccaaactccgccgtccaacgtgcgtactcccttccgcaagtgcgt
4E-T	Y10A, L15A	caaagattagtgccagggcctcgaaagtagacgcgttagctctaagatatgaag
	L36A, L39A	cgcaatgttcaacacgacttgaagcgcagacggcgggtttttggaaaattaatctgaa
	F41A, W42A, K43A	gacttgaattgcagacgctaggtgcggcggcgattaatctgaacacagctgcgtt
	Δ28–43	gccaaactccgccgtccaacgtgcgtactcccttccgcaagtgcgt
